# The Effect of Deworming on Growth in One-Year-Old Children Living in a Soil-Transmitted Helminth-Endemic Area of Peru: A Randomized Controlled Trial

**DOI:** 10.1371/journal.pntd.0004020

**Published:** 2015-10-01

**Authors:** Serene A. Joseph, Martín Casapía, Antonio Montresor, Elham Rahme, Brian J. Ward, Grace S. Marquis, Lidsky Pezo, Brittany Blouin, Mathieu Maheu-Giroux, Theresa W. Gyorkos

**Affiliations:** 1 McGill University, Department of Epidemiology, Biostatistics and Occupational Health, Montréal, Québec, Canada; 2 Research Institute of the McGill University Health Centre, Division of Clinical Epidemiology, Montréal, Québec, Canada; 3 Asociación Civil Selva Amazónica, Iquitos, Peru; 4 Department of Control of Neglected Tropical Diseases, World Health Organization, Geneva, Switzerland; 5 McGill University, Department of Medicine, Montréal, Québec, Canada; 6 JD MacLean Centre for Tropical Diseases, McGill University, Department of Medicine, Montréal, Québec, Canada; 7 National Reference Centre for Parasitology, Research Institute of the McGill University Health Centre, Montréal, Québec, Canada; 8 McGill University, School of Dietetics and Human Nutrition, Sainte Anne-de-Bellevue, Québec, Canada; 9 Department of Infectious Disease Epidemiology, Imperial College London, St. Mary’s Hospital, London, United Kingdom; Universidad San Francisco de Quito, ECUADOR

## Abstract

**Background:**

Appropriate health and nutrition interventions to prevent long-term adverse effects in children are necessary before two years of age. One such intervention may include population-based deworming, recommended as of 12 months of age by the World Health Organization in soil-transmitted helminth (STH)-endemic areas; however, the benefit of deworming has been understudied in early preschool-age children.

**Methodology/Principal Findings:**

A randomized, double-blind, placebo-controlled trial was conducted to determine the effect of deworming (500 mg single-dose crushed mebendazole tablet) on growth in one-year-old children in Iquitos, Peru. Children were enrolled during their routine 12-month growth and development clinic visit and followed up at their 18 and 24-month visits. Children were randomly allocated to: Group 1: deworming at 12 months and placebo at 18 months; Group 2: placebo at 12 months and deworming at 18 months; Group 3: deworming at both 12 and 18 months; or Group 4: placebo at both 12 and 18 months (i.e. control group). The primary outcome was weight gain at the 24-month visit. An intention-to-treat approach was used. A total of 1760 children were enrolled between September 2011 and June 2012. Follow-up of 1563 children (88.8%) was completed by July 2013. STH infection was of low prevalence and predominantly light intensity in the study population. All groups gained between 1.93 and 2.05 kg on average over 12 months; the average difference in weight gain (kg) compared to placebo was: 0.05 (95% CI: -0.05, 0.17) in Group 1; -0.07 (95%CI: -0.17, 0.04) in Group 2; and 0.04 (95%CI: -0.06, 0.14) in Group 3. There was no statistically significant difference in weight gain in any of the deworming intervention groups compared to the control group.

**Conclusions:**

Overall, with one year of follow-up, no effect of deworming on growth could be detected in this population of preschool-age children. Low baseline STH prevalence and intensity and/or access to deworming drugs outside of the trial may have diluted the potential effect of the intervention. Additional research is required to overcome these challenges and to contribute to strengthening the evidence base on deworming.

**Trial Registration:**

ClinicalTrials.gov (NCT01314937)

## Author Summary

The World Health Organization recommends starting population-based deworming interventions as of 12 months of age where intestinal worm infection is common; however, little is known about the benefits in early preschool-age children. We conducted a clinical trial to determine the effect of deworming on growth in one-year-old children in Peru. Participating children were randomly assigned to: 1) deworming at 12 months of age; 2) deworming at 18 months of age; 3) deworming at 12 and 18 months of age; or 4) no deworming (i.e. control group). A total of 1760 children were enrolled between September 2011 and June 2012, and followed up for one year. Overall, with one year of follow-up, no effect of deworming on growth could be detected in this population of preschool-age children. The potential benefit of the intervention may have been affected by low baseline infection prevalence and/or low compliance to the randomly assigned intervention. Additional research is required to overcome these challenges and to contribute to strengthening the evidence base on deworming.

## Introduction

The soil-transmitted helminth (STH) disease cluster includes *ascariasis*, *trichuriasis* and hookworm disease. It is considered to be one of the most common Neglected Tropical Diseases (NTD), affecting an estimated 1.45 billion people worldwide [1]. STHs are transmitted in contaminated food, water and the environment in areas of poverty in low- and middle-income countries. These intestinal parasites have a direct and indirect adverse impact on nutritional status by disrupting normal nutrient intake, excretion and utilization in their hosts and by causing blood loss and loss of appetite [2,3].

WHO recommends large-scale preventive chemotherapy programs, using anthelminthic treatment (i.e. deworming), for the high-risk groups of women of reproductive age, especially pregnant women, school-age children (i.e. 5 to 14 years of age), and preschool-age children (i.e. 1 to 4 years of age) in STH-endemic areas [4,5]. Adverse effects from deworming are infrequent, and when reported, are mild and transitory, including gastrointestinal upset and diarrhea [6]. Deworming interventions are often school-based in order to reach school-age children. In preschool-age children, deworming is often piggybacked onto vaccination or supplementation programs, child health days, or programs for the elimination of lymphatic filariasis [7]. However, preschool-age children lag behind their school-age counterparts as scaling-up of school-based programs continues while that of preschool programs remains a challenge [7]. The global proportion of at-risk preschool-age children receiving deworming in 2012 was estimated to be on the order of 25% [7]. This coverage has decreased since previous reports [8].

Prior to 2002, children under two years of age had been excluded from deworming interventions as the burden of STH infection was perceived to be low in this age group and the safety profile of available anthelminthics was not well established. In 2002, WHO convened an informal consultation of experts, and subsequently recommended the inclusion of children between 12 and 24 months of age in deworming activities using single-dose albendazole (in a reduced dose of 200 mg) or mebendazole (in the usual dose of 500 mg) [9]. These recommendations were based on animal studies, toxicity data and other safety data [10]. Despite the WHO recommendations and increasing evidence of the occurrence of STH infection in early preschool-age children [10–15], many countries still exclude children under 24 months of age from their national deworming programs. Providing evidence on the potential benefits of deworming in the younger age group between one and two years of age is essential. A study reviewing data from 54 countries confirmed that preventive interventions must occur during the first two years of life to prevent growth deficits, such as stunting and underweight [16]. Interventions at this time are essential to prevent both short- and longer-term adverse health effects [17]. The evidence-base on including deworming as one of the essential early childhood interventions in this critical window is, however, limited. Randomized controlled trials conducted exclusively in school-age children or in both preschool-age and school-age children have provided mixed evidence on deworming benefits on growth and development [6,18,19]. Few studies have focused exclusively on the preschool-age population [12,20–22]. There is some evidence that adverse consequences of even low prevalence and intensity STH infection may be more pronounced in children during this critical time period [11].

Considering the unique nutritional demands and growth patterns of younger children, aggregated results from older children do not provide a clear indication of the potential benefit of deworming on growth and nutrition in younger age groups. To fill this research gap, we therefore conducted a randomized controlled trial on the effect, and optimal timing and frequency, of a deworming intervention incorporated into routine child health services at one year of age. Our objective was to determine whether deworming would improve growth by two years of age.

## Methods

### Ethics approval and trial monitoring

This study received ethics approval in Peru from the Comité Institucional de Ética of the Universidad Peruana Cayetano Heredia and the Instituto Nacional de Salud, in Lima, and the local Ministry of Health office (Dirección Regional de Salud (DIRESA) Loreto) in Iquitos (S1 Text). Ethics approval was obtained in Canada from the Research Ethics Board of the Research Institute of the McGill University Health Centre in Montréal, Québec (S1 Text). An independent Data Safety and Monitoring Committee (DSMC) was established with three members, from Canada, the U.S., and Peru, to review all adverse events and approve continuation of the trial at three time points. At baseline, eligibility was assessed, and an informed consent form was signed by both parents or guardians of the child. In the case of a single parent (e.g. due to death, separation or divorce), only one signature was required. The trial was registered with ClinicalTrials.gov (NCT01314937). The CONSORT checklist is described in S1 Checklist and the trial protocol is described in S2 Text.

### Study design and enrollment procedures

We conducted a randomized, double-blind, parallel, placebo-controlled trial of a deworming intervention incorporated into routine growth and development (‘Crecimiento y Desarrollo” or CRED) visits in Iquitos, an STH-endemic area of the Peruvian Amazon. Details on baseline enrolment methodology and the study population have been described elsewhere [14]. Briefly, children were enrolled into the trial in their homes or participating health centres. Inclusion criteria were: 1) children attending any one of the 12 participating health centres for their 12-month CRED visit; and 2) children living in Belén, Iquitos, Punchana or San Juan districts. Exclusion criteria were: 1) children attending the health centre for suspected STH infection; 2) children who had received deworming treatment in the six months prior to the trial; 3) children whose families planned to move outside of the study area within the next 12 months; 4) children under 12 months of age or 14 months of age or older; and 5) children with any serious congenital or chronic medical condition. Any child who was excluded for medical reasons, and who was not already receiving regular health care, was referred to the health centre for follow-up by appropriate health personnel.

A baseline socio-demographic and epidemiological questionnaire (including family and child health and nutrition information) was administered in the home or health centre to the primary caregiver of the child. Baseline outcome measurements, including weight, length and the provision of a stool specimen, were ascertained in a subsequent visit in the health centre. All procedures were performed by dedicated, trained research assistants.

### Intervention groups

Following confirmation of eligibility, informed consent and all baseline outcome assessments in the health centres, children were randomized into one of four intervention groups:

Group 1 (MBD/PBO): Usual care and deworming at the 12-month CRED visit and usual care and placebo at the 18-month CRED visit.
Group 2 (PBO/MBD): Usual care and placebo at the 12-month CRED visit and usual care and deworming at the 18-month CRED visit.
Group 3 (MBD/MBD): Usual care and deworming at both the 12 and 18-month CRED visits.
Group 4 (PBO/PBO): Usual care and placebo at both the 12 and 18-month CRED visits (i.e. control group).

Deworming consisted of a single-dose mebendazole tablet (500 mg) (manufactured by Janssen Pharmaceuticals Inc.; donated by INMED Peru). The placebo was identical to the deworming tablet in terms of size, colour and markings (manufactured and purchased from Laboratorios Hersil, Peru). Tablets were crushed and mixed with juice for ease of administration and safety [23]. The crushed tablet was administered by research assistants at the end of each visit after all outcome assessments had been completed. All children received deworming at the 24-month visit according to Peruvian Ministry of Health guidelines [24]. Children received usual care interventions and services from health centre personnel [24]. This included the administration of measles, mumps and rubella (MMR) vaccination at the 12-month visit, and diphtheria, pertussis and tetanus (DPT) vaccine booster at the 18-month visit.

### Sample size

Sample size calculations were based on detecting the smallest meaningful difference among intervention groups in mean weight gain over 12 months, and took into account potential effect dilution from treating infected and non-infected children. From previous research in the study area, STH prevalence was expected to be 25% at 12 months of age [13]. To estimate expected growth, longitudinal growth data was collected from health centre registries in the study area in 2011. Mean weight gain ± standard deviation between 12 and 24 months in 100 untreated children was calculated to be 2.0 kg ± 0.8 kg. The sample size was calculated *a priori* such that comparisons could be made between all four groups to look at the overall effect of deworming, as well as the effect of timing and frequency of deworming.

In order to have 80% power to detect a minimum difference of 0.20 kg in mean weight gain among intervention groups, assuming a common standard deviation of 0.8 and a significance level of 0.05, and using a one-way ANOVA which accounts for pair-wise multiple comparisons between all groups (i.e. 6 comparisons) using the Tukey correction, the estimated sample size per group was 366 children. The required sample size was increased to 440 children per group (1760 in total), to take into account potential loss-to-follow-up of 20% after 12 months (based on attrition rates from previous studies in the area by the research team [25,26]) (MC4G Software©, GP Brooks, Ohio University, 2008).

### Randomization and masking

Computer-generated randomly ordered blocks of eight and twelve were used to randomly assign children to each intervention group in a 1:1:1:1 allocation ratio. Blocking ensured that the number of children assigned to each group would be balanced and reduced the potential for bias and confounding [27]. The random allocation sequence was generated by a biostatistician who was not otherwise involved in the trial. Research personnel not directly involved in the trial prepared small envelopes containing the randomly assigned intervention for each visit. These were numbered from 1 to 1760, with each number corresponding to one of the four intervention groups. Envelopes were stored in a temperature-regulated pharmacy at the research facility, and distributed by the Project Director (SAJ) or the local Study Coordinator (LP) in sequential order to research assistants until the sample size was achieved. Appropriate allocation concealment and randomly ordered block sizes ensured that the randomization sequence would not be predictable [27]. All health centre and research personnel, and parents of participants were blinded to intervention status.

### Follow-up visits

Children were followed-up at their 18 and 24-month visit in the health centre, at which time all outcome ascertainments were repeated. At the 18-month visit the second randomly assigned intervention was administered. Each visit was scheduled six months after the previous visit. In the case that a participant did not attend their 18-month visit, children remained eligible for the 24-month visit, which was scheduled 12 months after initial enrolment. If participants were not located prior to the day of their anticipated follow-up visit, or a scheduled date was missed, a minimum of four additional attempts were made to locate them. The original end dates of the 18-month follow-up and 24-month follow-up (i.e. trial completion) were each extended by one month (i.e. seven months and 13 months after the end of enrolment, respectively) to maximize follow-up rates. A monetary reimbursement was provided to cover travel costs for each visit.

### Outcome measurements

The pre-specified primary outcome measure was weight gain between the 12 and 24-month visit. Pre-specified secondary outcome measures were weight-for-age z-score, length gain, length-for-age z-score, change in STH infection prevalence and intensity, and change in development (i.e. cognitive, language and fine motor skills) between the 12 and 24-month visit. The development outcomes are reported separately.

Prior to commencing recruitment, in-depth practical training of the research assistants took place according to WHO guidelines [28,29] to ensure accurate outcome assessment and standardization. Inter and intra-rater reliability of over 95% was achieved for weight and length assessments, which are considered acceptable levels for anthropometric measurements [28,30].

Methods used for outcome measurements are described elsewhere [14]. Briefly, weight was measured using a portable electronic scale, accurate to the nearest 0.01 kg (Seca 334, Seca Corp., Baltimore, MD, USA). Length (i.e. the recommended measurement of height in children less than two years of age) was measured in duplicate as recumbent crown-heel length on a flat surface using a stadiometer (Seca 210, Seca Corp., Baltimore, MD, USA), accurate to the nearest millimetre. One stool specimen per child was collected to assess STH (e.g. *Ascaris*, *Trichuris* and hookworm) infection prevalence and intensity. For ethical reasons, only specimens from children receiving deworming treatment were immediately examined by trained laboratory technologists at the local research facility using the Kato-Katz method (single slide) for the presence and intensity (i.e. eggs per gram of feces) of STH infection [31]. At each time point, specimens from those children receiving placebo were stored at room temperature in 10% formalin and analyzed by the direct method for the presence of STH infection upon trial completion (Table 1).

**Table 1:** Randomly allocated treatment and corresponding analysis (method and timing) of stool specimens by group and visit.

Group	Visit	Treatment	Specimen analysis (method)	Specimen analysis (timing)
**1**	12-month	Mebendazole*	Kato-Katz	Immediate
	18-month	Placebo**	Direct	Stored, analyzed after 24-month visit
	24-month	Mebendazole	Kato-Katz	Immediate
**2**	12-month	Placebo	Direct	Stored, analyzed after 24-month visit
	18-month	Mebendazole	Kato-Katz	Immediate
	24-month	Mebendazole	Kato-Katz	Immediate
**3**	12-month	Mebendazole	Kato-Katz	Immediate
	18-month	Mebendazole	Kato-Katz	Immediate
	24-month	Mebendazole	Kato-Katz	Immediate
**4**	12-month	Placebo	Direct	Stored, analyzed after 24-month visit
	18-month	Placebo	Direct	Stored, analyzed after 24-month visit
	24-month	Mebendazole	Kato-Katz	Immediate

*single-dose 500 mg mebendazole tablet, crushed and mixed with juice

**single-dose tablet, identical in size, colour and markings to the mebendazole tablet

This approach ensured that children found to be infected were treated. This approach also aimed to minimize effect dilution which would have occurred if treatment had been provided to those found to be STH positive, but randomized to receive placebo. The Kato-Katz method is the recommended technique for assessment of the prevalence and intensity of intestinal parasitic infection in fresh stool [31]. For a one-stool specimen, sensitivity and specificity are over 96% for *Ascaris* and over 91% for *Trichuris* [32]. There is lower sensitivity and specificity for hookworm; however, hookworm infection is generally uncommon in very young children in this study area [13]. Additional details on the collection of stool specimens, including the ethical rationale for using two methods of analysis and how blinding was maintained, are published elsewhere [14]. Lower sensitivity to detect STH infection from storage and later analysis of specimens by the direct method was also anticipated [14].

A socio-demographic and epidemiological questionnaire was administered at each visit. At the follow-up visits, this included a question on whether deworming had been received between study visits (i.e. outside of the trial).

Information on minor and severe adverse events was obtained through passive reporting at follow-up visits or in between visits. Severe adverse events were based on WHO definitions and included: 1) death; 2) life-threatening conditions; 3) in-patient hospitalization or prolongation of an existing hospitalization; 4) persistent or significant disability/incapacity; 5) cancer; or 6) overdose (accidental or intentional) [5]. All reported illnesses that did not meet the definition of a serious adverse event were considered to be minor adverse events. All adverse events were reported to ethics committees. Summary reports of adverse events were also provided to the DSMC.

Data collection activities during fieldwork were regularly supervised by the Project Director (SAJ) and local Project Coordinator (LP). The consistency of egg count assessments was evaluated among the laboratory technologists using standard quality control methods [31]. The laboratory supervisor read 10% of the slides of the laboratory technologists without prior knowledge of the result to ensure quality control.

### Analyses

Weight-for-age z scores (WAZ) and length-for-age z scores (LAZ) were calculated using WHO Anthro software (Version 3, 2011). WHO categories were used to classify STH intensity according to species-specific counts of eggs per gram of feces (epg) [33]. Both arithmetic and geometric mean epg were calculated.

The primary outcome of the trial was mean weight gain in kilograms (kg) between the baseline 12-month visit and the 24-month follow-up visit (i.e. after 12 months). Mean weight gain (kg) was compared between the four intervention groups using unadjusted one-way ANOVA procedure. Secondary analyses which were specified *a priori* were conducted to examine differences between intervention groups in terms of change in derived weight indices (i.e. mean WAZ change) and length and derived length indices (mean length gain and mean LAZ change). Multivariable linear regression was also conducted adjusting for age, sex, socioeconomic status (based on an asset-based proxy index) [34,35] and continued breastfeeding at 12 months of age.

All analyses were first expressed using an intention-to-treat (ITT) approach such that participants were analyzed according to their assigned intervention group. Multiple imputation, using a Markov Chain Monte Carlo (MCMC) model with five imputations, was used for those who did not attend the 24-month follow-up visit. Variables related to the outcome, and hypothesized to be related to missing the follow-up visit(s) were used to impute missing weight and length measurements. These variables were baseline weight, length, socioeconomic status, continued breastfeeding at 12 months, sex, and age. Imputation was done separately by randomly assigned treatment group. Additional analyses were specified *a posteriori*, including: 1) using a complete case approach on all participants who had attended the final follow-up visit, 2) using a per-protocol approach excluding those participants who did not attend all three visits and/or who reported having received deworming outside of the trial between baseline and the final follow-up visit and 3) restricted to children positive for STH infection at baseline. These analyses were conducted for the following reasons: 1) complete case analyses were conducted for comparison purposes with intention-to-treat analyses with imputed data; 2) per-protocol analyses were conducted to account for higher than anticipated non-compliance to the assigned intervention; and 3) subgroup analysis in STH-infected children were conducted to account for the lower than anticipated baseline STH infection prevalence.

The primary research question on the effect of deworming was determined by comparing growth outcomes between each intervention group and the control group. To explore the secondary research question on the effect of the timing of deworming (i.e. at the 12-month visit or at the 18-month visit), growth outcomes in Group 1 were compared to Group 2. To explore the secondary research question on the effect of the frequency of deworming (i.e. provided once or twice), growth outcomes in Group 1 and Group 2 were each compared to Group 3. All three research questions were specified *a priori*.

The effect of deworming on STH indicators at 24 months was also examined using a generalized linear model with a log link, a Poisson distribution, and a robust variance estimator to estimate the risk ratio for the dichotomous outcomes of any STH infection, *Ascaris* infection, *Trichuris* infection and hookworm infection, where no infection (i.e. no STH infection, no *Ascaris* infection, no *Trichuris* infection and no hookworm infection, respectively) comprised the reference group.

All statistical analyses were performed using the Statistical Analysis Systems statistical software package version 9.3 (SAS Institute, Cary, NC, USA).

## Results

### Participant flow

**Fig 1:**
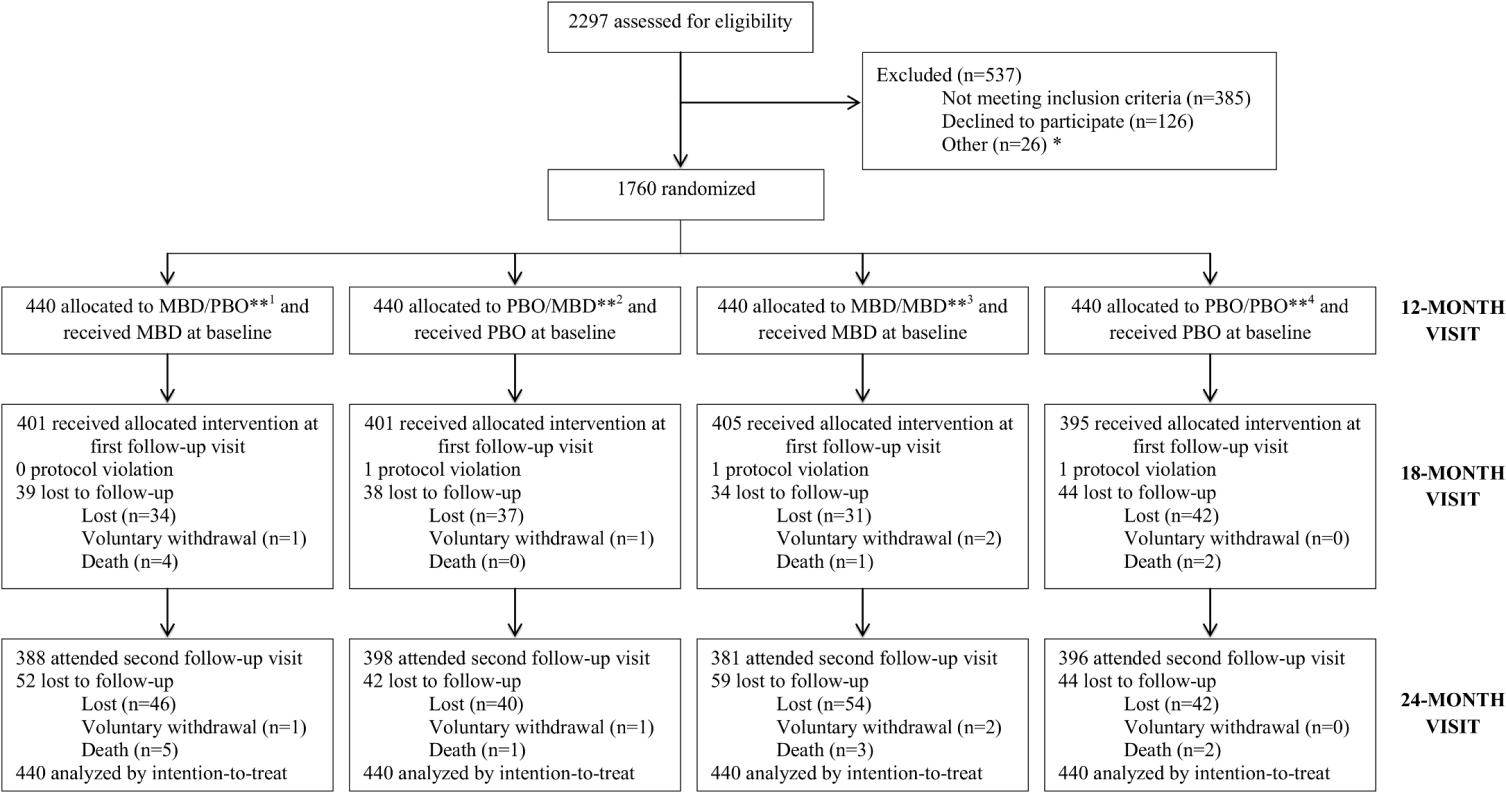
Trial profile. *26 participants were screened but were not enrolled once the sample size was met. **^1^ Group 1 (MBD/PBO) = mebendazole (12 months)/placebo (18 months); ^2^ Group 2 (PBO/MBD) = placebo (12 months)/mebendazole (18 months); ^3^ Group 3 (MBD/MBD) = mebendazole (12 months)/mebendazole (18 months); ^4^ Group 4 (PBO/PBO) = placebo (12 months)/placebo (18 months).

Between September 2011 and June 2012, the parents of 2297 children were approached to participate in the trial. Five-hundred and thirty-seven children were excluded as they did not meet the inclusion criteria (n = 385), declined to participate (n = 126), or were approached but not enrolled once the sample size was reached (n = 26). A total of 1760 children were randomized to the four groups (Fig 1). All children received the assigned intervention at baseline. A total of 1606 children (91.2%) attended their first follow-up at the 18-month visit between March 2012 and January 2013. Due to parental refusal, three children did not receive their randomly allocated intervention. The average time between the baseline and first follow-up visit was 6.3 months (± 0.41) and between the first follow-up visit and the second follow-up visit was 6.3 months (± 0.47). The average time between the baseline and second follow-up visit was 12.6 months (± 0.67). Time between visits was equivalent among intervention groups. The second follow-up visit was completed between September 2012 and July 2013.

### Compliance

A total of 1517 children (86.2%) attended all three visits. Of those who did not attend all three visits, 108 (6.1%) attended the first visit only, 89 children (5.1%) attended the first and second visits and 46 children (2.6%) attended the first and last visits. The proportion of children reported to have received deworming outside of the trial during the study period was 25.7% in Group 1; 26.8% in Group 2; 26.3% in Group 3; and 30.3% in Group 4. These differences were not statistically significant (p = 0.49).

### Characteristics of the study population

Baseline characteristics of the study population by intervention group are found in Table 2. Groups were similar in terms of baseline weight (kg) and length (cm), age (months), birth weight (kg) and length (cm), continued breastfeeding, up-to-date vaccinations and hospitalizations since birth. There were small differences in the proportion of girls in each group and vitamin A supplementation in the previous year. In terms of maternal and household characteristics, groups were similar in the proportion of mothers who were married or common-law, the level of maternal education, and access to potable water in the home. Small differences were found in maternal employment outside of the home and area of residence. Baseline characteristics were similar between children who attended the final follow-up visit and those who missed their final visit (S1 Table).

**Table 2:** Baseline characteristics of the study population (N = 1760) by intervention group, Iquitos, Loreto, Peru (September 2011-July 2013).

	MBD/PBO* ^1^	PBO/MBD * ^2^	MBD/MBD * ^3^	PBO/PBO* ^4^
	(n = 440)	(n = 440)	(n = 440)	(n = 440)
**Child characteristics**				
Weight [mean kg (SD**)]	8.6 (1.0)	8.8 (1.0)	8.7 (1.0)	8.7 (0.9)
Length [mean cm (SD)]	71.9 (2.4)	72.3 (2.4)	72.1 (2.5)	72.2 (2.5)
Age [mean months (SD)]	12.5 (0.4)	12.5 (0.5)	12.5 (0.4)	12.5 (0.5)
Birth weight [mean kg (SD)]	3.1 (0.5)	3.2 (0.5)	3.2 (0.5)	3.2 (0.5)
Birth length [mean cm (SD)]	49.2 (2.5)	49.5 (2.5)	49.4 (2.3)	49.5 (2.7)
Sex [n (%) female]	215 (48.9)	222 (50.5)	203 (46.1)	200 (45.5)
Continued breastfeeding at 12 months [n (%)]	394 (89.6)	395 (89.8)	394 (89.6)	392 (89.1)
Up-to-date vaccinations*** [n (%)]	346 (78.8)	351 (79.8)	358 (81.6)	355 (80.9)
Received vitamin A in previous year [n (%)]	213 (48.4)	241 (54.8)	251 (57.1)	216 (49.1)
Hospitalizations since birth [n (%)]	402 (91.4)	397 (90.2)	402 (91.4)	396 (90.0)
Walking without support [n (%)]	111 (25.2)	104 (23.7)	117 (26.6)	101 (23.1)
**Maternal characteristics**				
Married or common-law [n (%)]	358 (81.4)	351 (79.8)	357 (81.1)	357 (81.1)
Secondary education completed [n (%)]	142 (32.4)	140 (31.8)	133 (30.2)	139 (31.6)
Employment outside the home [n (%)]	47 (10.7)	45 (10.2)	50 (11.4)	37 (8.4)
**Household characteristics**				
Peri-urban or rural residence [n (%)]	382 (86.8)	391 (88.9)	388 (88.2)	399 (90.7)
Potable water in home [n (%)]	230 (52.3)	218 (49.6)	230 (52.3)	220 (50.0)
Earth or wood house material [n (%)]	342 (77.7)	342 (77.7)	338 (76.8)	332 (75.5)

*^1^Group 1 (MBD/PBO) = mebendazole at the 12-month visit and placebo at the 18-month visit

^2^Group 2 (PBO/MBD) = placebo at the 12-month visit and mebendazole at the 18-month visit

^3^Group 3 (MBD/MBD) = mebendazole at the 12 and 18-month visit

^4^Group 4 (PBO/PBO) = placebo at the 12 and 18-month visit

**SD = standard deviation

***Up-to-date vaccinations include those scheduled between birth and 11 months of age (i.e. one dose of Bacille Calmette-Guérin (BCG), one dose of hepatitis B, three doses of polio, three doses of pentavalent, two doses of rotavirus, and two doses of pneumococcal)

At baseline, the prevalence of any STH infection was 14.5% in the two groups whose specimens were analyzed by the Kato-Katz method (i.e. 13.6% in MBD/PBO and 15.2% in MBD/MBD) (Table 3). At the 18-month visit, any STH prevalence was 28.5% (i.e. 30.7% in PBO/MBD and 26.4% in MBD/MBD). As expected due to lower sensitivity, STH prevalence in children whose stool specimens were analyzed by the direct method at 12 and 18 months was moderately lower (i.e. 10.5% and 24.5%, respectively). Certain sensitivity analyses were therefore conducted in subgroups of children found to be STH-positive 1) by both the direct and Kato-Katz methods and 2) only by the Kato-Katz method. Despite potential misclassification of STH infection status in children whose specimens were analyzed by the direct method, this strategy allowed for maximum comparison among all groups. Infection was predominantly low intensity for *Trichuris* and hookworm infection at all three time points; however, moderate and heavy intensity *Ascaris* infection increased over the one-year follow-up period (Table 3).

At the 24-month visit, at which time all specimens were analyzed by the Kato-Katz method, the overall prevalence of any STH increased to 42.6%. Prevalence of *Ascaris*, *Trichuris* and any STH infection was moderately lower in the groups which received deworming at the 18-month visit. Hookworm infection remained negligible. No statistically significant difference in any STH prevalence or *Ascaris* or hookworm prevalence was observed in any of the deworming intervention groups compared to the control group; however, a statistically significantly lower prevalence of *Trichuris* infection was observed in Group 3, which received mebendazole at both the 12 and 18-month visits, compared to the control group (RR = 0.69; 95% CI: 0.52, 0.90) (S2 Table).

**Table 3:** Soil-transmitted helminth (STH) infection prevalence and intensity at the a) 12-month (n = 880)* ^1^, b) 18-month (n = 807)* ^2^ and c) 24-month (n = 1563)* ^3^ follow-up visits by intervention group, Iquitos, Loreto, Peru (September 2011-July 2013).

	a) 12-month visit	b) 18-month visit
	MBD/PBOǂ ^1^ (n = 440)	MBD/MBDǂ ^3^ (n = 440)	PBO/MBDǂ ^2^ (n = 401)	MBD/MBDǂ ^3^ (n = 405)
*Ascaris lumbricoides*				
Prevalence (#, %)	48 (10.9)	52 (11.8)	93 (23.2)	82 (20.2)
Intensity				
No (#, %)	392 (89.1)	388 (88.2)	308 (76.8)	323 (79.8)
Light (#, %)	40 (9.1)	46 (10.4)	73 (18.2)	56 (13.8)
Moderate (#, %)	8 (1.8)	6 (1.4)	17 (4.2)	25 (6.2)
Heavy (#, %)	0 (0.0)	0 (0.0)	3 (0.8)	1 (0.2)
AM† (95% CI)	321.2 (171.4, 471.1)	253.9 (144.6, 363.2)	1524.6 (668.2, 2381.1)	1227.7 (687.9, 1767.5)
GM§ (95% CI)	2.2 (1.8, 2.8)	2.2 (1.8, 2.8)	5.4 (3.9, 7.3)	4.5 (3.3, 6.1)
*Trichuris trichiura*				
Prevalence (#, %)	17 (3.9)	22 (5.0)	55 (13.7)	44 (10.8)
Intensity				
No (#, %)	423 (96.2)	418 (95.0)	346 (86.3)	361 (89.2)
Light (#, %)	16 (3.6)	20 (4.6)	52 (13.0)	41 (10.1)
Moderate (#, %)	1 (0.2)	2 (0.4)	3 (0.7)	3 (0.7)
AM (95% CI)	18.0 (-4.4, 40.5)	15.1 (3.8, 26.4)	41.5 (9.7, 73.2)	30.8 (10.6, 51.0)
GM (95% CI)	1.2 (1.1, 1.3)	1.3 (1.2, 1.4)	1.9 (1.6, 2.2)	1.7 (1.5, 2.0)
Hookworm				
Prevalence (#, %)	3 (0.7)	2 (0.5)	1 (0.3)	6 (1.5)
Intensity				
No (#, %)	437 (99.3)	438 (99.5)	400 (99.8)	399 (98.5)
Light (#, %)	3 (0.7)	2 (0.5)	1 (0.2)	6 (1.5)
AM (95% CI)	1.4 (-0.9, 3.7)	0.7 (-0.5, 1.8)	1.5 (-1.4, 4.4)	3.6 (-0.7, 7.9)
GM (95% CI)	1.03 (1.0, 1.1)	1.0 (1.0, 1.0)	1.0 (1.0, 1.0)	1.1 (1.0, 1.1)
Any STH infection				
Prevalence (#, %)	60 (13.6)	67 (15.2)	123 (30.7)	107 (26.4)
**c) 24-month visit**	MBD/PBOǂ ^1^ (n = 388)	PBO/MBDǂ ^2^ (n = 398)	MBD/MBDǂ ^3^ (n = 381)	PBO/PBOǂ ^4^ (n = 396)
*Ascaris lumbricoides*				
Prevalence (#, %)	128 (33.0)	127 (31.9)	117 (30.7)	128 (32.3)
Intensity				
No (#, %)	260 (67.0)	271 (68.1)	264 (69.3)	268 (67.7)
Light (#, %)	85 (21.9)	88 (22.1)	82 (21.5)	88 (22.2)
Moderate (#, %)	40 (10.3)	37 (9.3)	33 (8.7)	38 (9.6)
Heavy (#, %)	3 (0.8)	2 (0.5)	2 (0.5)	2 (0.5)
AM (95% CI)	2246.7 (1491.9, 3001.4)	2442.5 (948.3, 3936.7)	2205.5 (1179.9, 3231.2)	1952.0 (1238.8, 2665.2)
GM (95% CI)	12.1 (8.3, 17.4)	10.7 (7.5, 15.2)	9.5 (6.7, 13.5)	10.3 (7.3, 14.6)
*Trichuris trichiura*				
Prevalence (#, %)	100 (25.8)	83 (20.9)	68 (17.9)	103 (26.0)
Intensity				
No (#, %)	288 (74.2)	315 (79.1)	313 (82.2)	293 (74.0)
Light (#, %)	97 (25.0)	82 (20.6)	66 (17.3)	100 (25.3)
Moderate (#, %)	3 (0.8)	1 (0.3)	2 (0.5)	3 (0.7)
AM (95% CI)	57.5 (31.0, 84.0)	26.4 (16.8, 35.9)	34.1 (16.5, 51.8)	55.6 (36.8, 74.3)
GM (95% CI)	3.3 (2.7, 4.1)	2.5 (2.1, 2.9)	2.2 (1.9, 2.7)	3.4 (2.8, 4.2)
Hookworm				
Prevalence (#, %)	4 (1.0)	6 (1.5)	5 (1.3)	9 (2.3)
Intensity				
No (#, %)	384 (99.0)	392 (98.5)	376 (98.7)	387 (97.7)
Light (#, %)	4 (1.0)	6 (1.5)	5 (1.3)	9 (2.3)
AM (95% CI)	1.4 (-0.6, 3.4)	1.6 (-0.2, 3.3)	2.1 (-0.4, 4.7)	7.5 (-2.2, 17.2)
GM (95% CI)	1.0 (1.0, 1.1)	1.1 (1.0, 1.1)	1.1 (1.0, 1.1)	1.1 (1.0, 1.2)
Any STH infection				
Prevalence (#, %)	175 (45.1)	163 (41.0)	149 (39.1)	179 (45.2)

* STH results at all visits include only children whose specimens were analyzed by the Kato-Katz method (i.e. ^1^Group 1 and Group 3 at 12-month visit; ^2^Groups 2 and 3 at 18-month visit (results were not available for 73 children who were lost to follow-up); ^3^All groups at the 24-month visit (results were not available for 197 children who were lost to follow-up))

ǂ^1^Group 1 (MBD/PBO) = mebendazole at the 12-month visit and placebo at the 18-month visit

^2^Group 2 (PBO/MBD) = placebo at the 12-month visit and mebendazole at the 18-month visit

^3^Group 3 (MBD/MBD) = mebendazole at the 12 and 18-month visit

^4^Group 4 (PBO/PBO) = placebo at the 12 and 18-month visit

†AM = arithmetic mean eggs per gram

§GM = geometric mean eggs per gram. A value of 1 was added to each observation to calculate the geometric mean.

The prevalence of stunting and underweight increased from 24.2% and 8.6% at baseline to 46.8% and 10.2%, respectively, at the 24-month visit.

### Overall effect of deworming on primary and secondary anthropometric outcomes

All groups gained between 1.93 and 2.05 kg in weight and between 9.61 and 9.84 cm in length, on average over 12 months. The greatest changes in all growth outcomes between the 12- and 24-month visits were seen in Group 1 (Table 4). The average difference in weight gain (kg) compared to placebo was: 0.05 (95% CI: -0.05, 0.17) in Group 1; -0.07 (95%CI: -0.17, 0.04) in Group 2; and 0.04 (95%CI: -0.06, 0.14) in Group 3. When comparing the outcomes in each of the deworming intervention groups to the control group, however, no statistically significant effect was detected in unadjusted or adjusted ITT analysis (Table 4). No statistically significant difference in any intervention group compared to the control group was seen in per-protocol analysis (S3 Table), complete case analysis (S4 Table) or in analysis restricted to only those children who were positive for STH infection at baseline (S5 Table).

**Table 4:** Overall primary effect of deworming on anthropometric outcomes over 12 months, using one-way ANOVA and multivariable linear regression analyses (N = 1760*), Iquitos, Loreto, Peru (September 2011 –July 2013).

	MBD/PBO** ^1^	PBO/MBD** ^2^	MBD/MBD** ^3^	PBO/PBO** ^4^
	(n = 440)	(n = 440)	(n = 440)	(n = 440)
**Primary outcome**				
Weight gain, kg	2.05	1.93	2.04	2.00
(95% CI)	(1.98, 2.13)	(1.85, 2.02)	(1.97, 2.11)	(1.93, 2.06)
Unadjusted difference	0.05	-0.07	0.04	reference
(95% CI)	(-0.05, 0.16)	(-0.17, 0.04)	(-0.06, 0.14)	
p-value	0.322	0.217	0.442	
Adjustedǂ difference	0.06	-0.06	0.05	reference
(95% CI)	(-0.05, 0.17)	(-0.16, 0.04)	(-0.05, 0.15)	
p-value	0.289	0.259	0.318	
**Secondary outcomes**				
Length gain, cm	9.84	9.53	9.67	9.61
(95% CI)	(9.64, 10.05)	(9.33, 9.74)	(9.50, 9.85)	(9.41, 9.81)
Unadjusted difference	0.23	-0.08	0.06	reference
(95% CI)	(-0.07, 0.53)	(-0.38, 0.23)	(-0.21, 0.34)	
p-value	0.129	0.626	0.651	
Adjusted difference	0.26	-0.06	0.12	reference
(95% CI)	(-0.04, 0.55)	(-0.36, 0.25)	(-0.15, 0.39)	
p-value	0.087	0.714	0.390	
WAZ†^1^ change	-0.23	-0.36	-0.24	-0.28
(95% CI)	(-0.30, -0.16)	(-0.43, -0.29)	(-0.30, -0.18)	(-0.34, -0.22)
Unadjusted difference	0.05	-0.08	0.04	reference
(95% CI)	(-0.05, 0.14)	(-0.17, 0.01)	(-0.05, 0.13)	
p-value	0.321	0.090	0.359	
Adjusted difference	0.05	-0.07	0.04	reference
(95% CI)	(-0.04, 0.15)	(-0.16, 0.02)	(-0.05, 0.13)	
p-value	0.277	0.126	0.339	
LAZ†^2^ change	-0.51	-0.64	-0.56	-0.59
(95% CI)	(-0.58, -0.44)	(-0.71, -0.57)	(-0.62, -0.49)	(-0.66, -0.52)
Unadjusted difference	0.07	-0.05	0.03	reference
(95% CI)	(-0.02, 0.17)	(-0.15, 0.05)	(-0.06, 0.13)	
p-value	0.132	0.328	0.474	
Adjusted difference	0.09	-0.04	0.04	reference
(95% CI)	(-0.01, 0.18)	(-0.14, 0.06)	(-0.05, 0.13)	
p-value	0.083	0.454	0.377	

Results are expressed as mean (95% Confidence Interval)

* Intention-to-treat analysis includes data from 1563 children for whom final outcome information was available, and 197 children who were lost to follow-up and whose outcome information was estimated using multiple imputation

**^1^Group 1 (MBD/PBO) = mebendazole at the 12-month visit and placebo at the 18-month visit

^2^Group 2 (PBO/MBD) = placebo at the 12-month visit and mebendazole at the 18-month visit

^3^Group 3 (MBD/MBD) = mebendazole at the 12 and 18-month visit

^4^Group 4 (PBO/PBO) = placebo at the 12 and 18-month visit

ǂ Adjusted models include age, sex, socioeconomic status and continued breastfeeding at 12 months of age

†^1^WAZ = weight-for-age z score

^2^LAZ = length-for-age z score. Z scores were derived using WHO international growth standards [36]

### Effect of deworming timing on primary and secondary anthropometric outcomes

In examining the effect of the timing at which deworming was administered, a statistically significant improvement was seen in Group 1 compared to Group 2, in terms of weight gain (unadjusted difference 0.12 kg; 95% CI: 0.01; 0.23), length gain (unadjusted difference 0.31 cm; 95% CI: 0.04, 0.58), WAZ change (unadjusted difference 0.13; 95% CI: 0.03, 0.23), and LAZ change (unadjusted difference: 0.12; 95% CI: 0.03, 0.21) between baseline and the final follow-up visit in unadjusted analyses (S6 Table). These results remained significant in adjusted analyses (S6 Table) per-protocol analysis (S7 Table), complete case analysis (S8 Table). In subgroup analyses restricted to children positive for STH infection at baseline, no significant differences were observed between groups (S9 Table).

### Effect of deworming frequency on primary and secondary anthropometric outcomes

In comparing the difference in anthropometric outcomes between Group 1, receiving deworming once yearly, and Group 3, receiving deworming twice yearly, no additional benefit on weight or length was apparent for twice-yearly deworming in unadjusted or adjusted analyses (S10 Table). Results remained consistent in per-protocol analysis (S11 Table), complete case analysis (S12 Table) and in restricted analyses to children infected with STH at baseline (S13 Table). A statistically significant benefit, however, was observed in Group 3 compared to Group 2, in terms of weight gain and WAZ change. These results remained significant for both weight gain and WAZ change when adjusting for baseline characteristics, in per-protocol and complete case analyses.

### Adverse events

From baseline until the end of follow-up, 38 minor adverse events were reported and were similarly distributed among groups (i.e. Group 1: 7; Group 2: 10; Group 3: 12; and Group 4: 9). There were 18 serious adverse events reported: Group 1: 5 deaths and 2 hospitalizations; Group 2: 1 death and 0 hospitalizations; Group 3: 3 deaths and 2 hospitalizations; and Group 4: 2 deaths and 3 hospitalizations. Ten serious adverse events occurred after administration of mebendazole (i.e. 7 deaths and 3 hospitalizations) and eight serious adverse events occurred after administration of placebo (i.e. 4 deaths and 4 hospitalizations). The range of time between administration of the randomized intervention and occurrence of the serious adverse event was 6 days to 6 months for hospitalizations and 18 days and 7 months for deaths. None of these serious adverse events were deemed to be related to the deworming intervention by the DSMC, Research Ethics Committees in Canada and Peru, or the trial investigators.

## Discussion

This is the largest double-blind, randomized, placebo-controlled trial of deworming to our knowledge that has been conducted exclusively in children during the second year of life. This is the age at which WHO first recommends starting mass deworming programs, and it is also a time of rapid growth, development and STH acquisition. Our trial had several strengths. These include: 1) its randomized controlled design, which minimized confounding and the influence of external factors; 2) a large sample size, so that primary analyses were sufficiently powered; 3) a high follow-up rate, despite a highly mobile population and environmental challenges such as flooding which displaced many participants in the study area; 4) consistency of results in intention-to-treat, complete case and per-protocol analyses, demonstrating that results from children attending the final study visit are likely generalizable to the original trial population; and 5) community-based canvassing in the study area prior to recruitment to attempt to reach the entire study population [14].

### Overall effect of deworming

We were not able to demonstrate an overall benefit on the primary research question of deworming on growth between any of the intervention groups compared to the control group after one year of follow-up in intention-to-treat analysis or in further sensitivity analyses. Our results are consistent with a recent cluster-randomized trial of albendazole (administered every six months to children from six months to six years of age) conducted in north India where light intensity STH infection was also predominant [21]. It is also consistent with a study in Uganda in children aged 15 months to 5 years who received quarterly albendazole [22]. Our findings do, however, contradict other trials in preschool-age children that found a positive effect of deworming on growth indicators [12,20].

The lack of benefit in our study compared to these other studies could reflect a true lack of effect of deworming on growth in the time period and/or study population. This population of children has a high level of malnutrition that may not be able to be treated solely by one or two doses of deworming in a one-year time period. In addition, we were not able to demonstrate a statistically significant reduction in any STH or species-specific prevalence with any of the deworming interventions, except for a reduction in *Trichuris* infection with twice-yearly deworming. The poor effect of the deworming intervention on STH prevalence measured after 6 and 12 months was almost certainly influenced by the dynamics of re-infection and new infection occurring between study time points. Future studies, which are adequately powered to detect changes in STH infection over time, are needed to confirm these findings.

If there were, however, a true effect that was not observed, the short follow-up time may have limited the potential to detect this benefit. It is likely that, in the one year period of our study, a steady state has not yet been achieved, in terms of either STH infection (e.g. as evidenced by the over threefold increase in STH prevalence from 12 to 24 months of age) or growth (e.g. as evidenced by a negative deviation of WAZ and LAZ compared to the international WHO growth standard over 12 months). Benefits of the deworming intervention may be apparent only with a longer follow-up time.

The low prevalence and intensity of infection, in particular, may have limited the impact of deworming, which reduces morbidity primarily through a reduction in moderate and heavy intensity infection. In deworming interventions, nutritional improvements are not a direct consequence of drug administration but a result of the elimination of parasites that are competing for nutrients. When the intervention is administered to a population with low prevalence and/or intensity, the short-term benefits could be difficult to measure. WHO recommends the periodic (once or twice-yearly) administration of antihelminthics as a means of controlling morbidity from STH infection. The nutritional benefits are a consequence of the maintenance of very a low level of these infections in childhood.

The baseline prevalence of STH infection in the study population was lower than had been anticipated based on a study conducted in the area just three to four years prior (i.e. in 2007 and 2008) [13]. The number of children who could have potentially benefited from deworming in the trial was therefore reduced, resulting in a reduction in power to detect an effect of the expected size. Results from a previous trial suggested that deworming could improve growth in young children, even with low intensity infection [12]; however, we did not observe this in our trial. Preventive chemotherapy programs include treatment of both infected and uninfected children; nonetheless, our results suggest that research studies should be conducted in areas of high STH prevalence to ensure as little effect dilution as possible. With increasing implementation of deworming programs, a rapid assessment in the age group and study area to determine baseline prevalence and intensity may be warranted before beginning any research study.

### Effect of deworming timing and frequency

Our trial was unique in using a multiple group design to look additionally at the secondary research questions of differences in the timing and frequency among the groups that received deworming. Such considerations are important in operationalizing deworming interventions in this age group. Our results suggest that, if deworming is provided between one and two years of age, there is a significant benefit of providing it earlier rather than later. Our results also demonstrate that there was no added benefit from an additional dose provided at 18 months of age (over and above that at 12 months of age). These results were consistent in unadjusted and adjusted analysis, as well as in sensitivity analyses, for multiple growth indicators. A true benefit of earlier deworming compared to later deworming is biologically plausible, as suggested in nutritional research showing the importance of incorporating interventions as early as possible to prevent adverse health and nutritional consequences [16]. However, in light of the lack of benefit of any of the deworming interventions compared to the control group and the low STH prevalence and intensity at baseline, these results should be interpreted with caution. The difference in growth between Groups 1 and 2 may be due to a shorter follow-up from the time of intervention to the time of outcome measurement (i.e. 12 months in Group 1 vs. 6 months in Group 2), or a chance finding of lower average weight gain in Group 2 compared to all three other groups. Although the number of statistically significant findings was more than due to chance alone, and all comparisons, except for sensitivity analyses (i.e. complete case, per-protocol, and subgroup analyses in STH-infected individuals), were specified *a priori*, we cannot rule out the possibility that this difference could be a spurious finding due to chance alone.

### Compliance

One issue that arose after beginning the study was difficulty with compliance, as over 25% of children received deworming at least once outside of the assigned intervention group. A higher proportion of children in the control group had been reported to have received deworming outside of the trial, but this proportion was not statistically significantly different from the other groups. Even after taking non-compliance into account by conducting a per-protocol analysis, excluding those who took deworming outside of the trial and/or who did not attend all three study visits, no statistically significant difference in growth outcomes was observed. The ease of access to deworming was an unexpected result as deworming is not routinely provided to children under two years of age in the study area; however, this level of access to deworming outside of the study protocol has been observed in other trials in preschool-age children [22]. With the growing presence of deworming campaigns and availability of antihelminthics without a prescription in many countries, this finding of non-compliance will likely become increasingly common. Although for ethical and logistical purposes we could not restrict access to antihelminthics outside of the trial, it is imperative that compliance is measured in all deworming research studies and taken into account in the analysis of results.

### Stool specimen analysis

For ethical and scientific reasons, we did not immediately analyze stool specimens from children randomized to placebo at the 12 or 18-month visits. This meant that accurate STH prevalence and intensity were not available for those receiving placebo (i.e. Group 2 (PBO/PBO) and Group 3 (MBD/MBD) at 12 months, and Group 1 (MBD/PBO) and Group 3 (MBD/MBD) at 18 months), and that results from the Kato-Katz and direct methods were not easily comparable (Table 1). Examining a single stool specimen with a single technique (i.e. Kato-Katz) may have also decreased the sensitivity to detect STH infection, particularly in those with low intensity infection [37]; however, considering the sample size and age group of children in the study, the collection of multiple specimens was not considered to be feasible. Our strategy had the advantage of providing accurate overall baseline STH prevalence of the study population (as all groups would be expected to have similar baseline prevalences due to randomization), and accurate final STH prevalences at the 24-month visit (at which time all groups were analyzed by the Kato-Katz method). As STH infection status was a secondary outcome (weight gain being the primary outcome), misclassification of infection status would not have affected the analyses on growth or on the effect of the deworming intervention on STH infection at the 24-month visit. Misclassification might have affected only the secondary subgroup analyses restricted to the STH-infected population. Despite the limitations to the strategy we employed, we consider ours to have methodological advantages to other strategies which have been used, such as: 1) not collecting stool specimens, which provides no information on baseline or follow-up STH infection[20], or; 2) analyzing all stool specimens immediately, and treating those found to be infected, regardless of allocated intervention group, which would dilute the effect size by providing treatment to those randomized to placebo if found to be STH-positive [22].

### Future research focus and directions

Overall, this is the first trial to provide evidence on the effect of deworming, including optimal timing and frequency, on growth exclusively in children in the critical time window between one and two years of age. This trial demonstrated the feasibility of incorporating deworming into routine growth and development health clinics along with other essential early childhood interventions. We were also able to demonstrate safety of the deworming intervention in this age group, with similar numbers of serious adverse events occurring after mebendazole and placebo administration. This is consistent with results from previous studies [22,23,38]. Continued observational follow-up of the trial cohort is currently taking place, and will provide evidence on the longer-term effects of deworming up to five years of age.

Future studies looking at the benefit of deworming on growth in this age group should include study areas of higher STH prevalence and/or intensity, higher potential compliance to the assigned intervention (i.e. lower availability of anthelminthics in the community) and longer follow-up time. Further studies should include other factors that are important to consider for scaling up deworming interventions in this age group. This includes: 1) cost-effectiveness of preventive chemotherapy vs. analyzing and treating only infected individuals; 2) feasibility and cost-effectiveness of integrating deworming with other health, nutritional and environmental interventions, particularly health education and micronutrient supplementation; 3) health and nutritional consequences of low intensity infection in younger age groups; and 4) inclusion of high-risk children living in more remote areas and/or those who do not regularly attend health services. This type of research is essential to contribute to strengthening the evidence base on deworming.

## Supporting Information

S1 Checklist CONSORT checklist. (DOC)

S1 Text Ethics approvals. (PDF)

S2 Text Protocol. (PDF)

S1 Table Baseline characteristics of children who attended the 24-month visit (n = 1563) compared to those who did not attend the 24-month visit (n = 197). (DOCX)

S2 Table Effect of deworming on any STH and species-specific prevalence over 12 months, using a generalized linear model, complete case analysis (n = 1563). (DOCX)

S3 Table Overall effect of deworming on anthropometric outcomes over 12 months, using one-way ANOVA and multivariable linear regression analysis, per-protocol analysis (n = 1103). (DOCX)

S4 Table Overall effect of deworming on anthropometric outcomes over 12 months, using one-way ANOVA and multivariable linear regression analysis, complete case analysis (n = 1563). (DOCX)

S5 Table Overall effect of deworming on anthropometric outcomes over 12 months, using one-way ANOVA and multivariable linear regression analysis, restricted to STH-infected children at baseline (n = 186). (DOCX)

S6 Table The effect of timing of deworming on anthropometric outcomes over 12 months, using one-way ANOVA and multivariable linear regression analysis, intention-to-treat analysis (n = 880). (DOCX)

S7 Table The effect of timing of deworming on anthropometric outcomes over 12 months, using one-way ANOVA and multivariable linear regression analysis, per-protocol analysis (n = 561). (DOCX)

S8 Table The effect of timing of deworming on anthropometric outcomes over 12 months, using one-way ANOVA and multivariable linear regression analysis, complete case analysis (n = 786). (DOCX)

S9 Table The effect of timing of deworming on anthropometric outcomes over 12 months, using one-way ANOVA and multivariable linear regression analysis, restricted to STH-infected children at baseline (n = 98). (DOCX)

S10 Table The effect of frequency of deworming on anthropometric outcomes over 12 months, using one-way ANOVA and multivariable linear regression analysis, intention-to-treat analysis (n = 1320). (DOCX)

S11 Table The effect of frequency of deworming on anthropometric outcomes over 12 months, using one-way ANOVA and multivariable linear regression analysis, per-protocol analysis (n = 836). (DOCX)

S12 Table The effect of frequency of deworming on anthropometric outcomes over 12 months, using one-way ANOVA and multivariable linear regression analysis, complete case analysis (n = 1167). (DOCX)

S13 Table The effect of frequency of deworming on anthropometric outcomes over 12 months, using one-way ANOVA and multivariable linear regression analysis, restricted to STH-infected children at baseline (n = 154). (DOCX)
