# Correction: The Effect of Deworming on Growth in One-Year-Old Children Living in a Soil-Transmitted Helminth-Endemic Area of Peru: A Randomized Controlled Trial

**DOI:** 10.1371/journal.pntd.0004288

**Published:** 2015-12-04

**Authors:** The PLOS Neglected Tropical Diseases Staff

There are several errors in Table 3, including the formatting of the headings. Please see the complete, correct Table 3 here.

**Table 1:** Soil-transmitted helminth (STH) infection prevalence and intensity at the a) 12-month (n = 880)* ^1^, b) 18-month (n = 806)* ^2^ and c) 24-month (n = 1563)* ^3^ follow-up visits by intervention group, Iquitos, Loreto, Peru (September 2011-July 2013).

	**a) 12-month visit**		**b) 18-month visit**	
	MBD/PBOǂ ^1^ (n = 440)	MBD/MBDǂ ^3^ (n = 440)	PBO/MBDǂ ^2^ (n = 401)	MBD/MBDǂ ^3^ (n = 405)
*Ascaris lumbricoides*				
Prevalence (#, %)	48 (10.9)	52 (11.8)	93 (23.2)	82 (20.2)
Intensity				
No (#, %)	392 (89.1)	388 (88.2)	308 (76.8)	323 (79.8)
Light (#, %)	40 (9.1)	46 (10.4)	73 (18.2)	56 (13.8)
Moderate (#, %)	8 (1.8)	6 (1.4)	17 (4.2)	25 (6.2)
Heavy (#, %)	0 (0.0)	0 (0.0)	3 (0.8)	1 (0.2)
AM† (95% CI)	321.2 (171.4, 471.1)	253.9 (144.6, 363.2)	1524.6 (668.2, 2381.1)	1227.7 (687.9, 1767.5)
GM§ (95% CI)	2.2 (1.8, 2.8)	2.2 (1.8, 2.8)	5.4 (3.9, 7.3)	4.5 (3.3, 6.1)
*Trichuris trichiura*				
Prevalence (#, %)	17 (3.9)	22 (5.0)	55 (13.7)	44 (10.8)
Intensity				
No (#, %)	423 (96.2)	418 (95.0)	346 (86.3)	361 (89.2)
Light (#, %)	16 (3.6)	20 (4.6)	52 (13.0)	41 (10.1)
Moderate (#, %)	1 (0.2)	2 (0.4)	3 (0.7)	3 (0.7)
AM (95% CI)	18.0 (-4.4, 40.5)	15.1 (3.8, 26.4)	41.5 (9.7, 73.2)	30.8 (10.6, 51.0)
GM (95% CI)	1.2 (1.1, 1.3)	1.3 (1.2, 1.4)	1.9 (1.6, 2.2)	1.7 (1.5, 2.0)
Hookworm				
Prevalence (#, %)	3 (0.7)	2 (0.5)	1 (0.3)	6 (1.5)
Intensity				
No (#, %)	437 (99.3)	438 (99.5)	400 (99.8)	399 (98.5)
Light (#, %)	3 (0.7)	2 (0.5)	1 (0.2)	6 (1.5)
AM (95% CI)	1.4 (-0.9, 3.7)	0.7 (-0.5, 1.8)	1.5 (-1.4, 4.4)	3.6 (-0.7, 7.9)
GM (95% CI)	1.03 (1.0, 1.1)	1.0 (1.0, 1.0)	1.0 (1.0, 1.0)	1.1 (1.0, 1.1)
Any STH infection				
Prevalence (#, %)	60 (13.6)	67 (15.2)	123 (30.7)	107 (26.4)
**c) 24-month visit**	MBD/PBOǂ ^1^ (n = 388)	PBO/MBDǂ ^2^ (n = 398)	MBD/MBDǂ ^3^ (n = 381)	PBO/PBOǂ ^4^ (n = 396)
*Ascaris lumbricoides*				
Prevalence (#, %)	128 (33.0)	127 (31.9)	117 (30.7)	128 (32.3)
Intensity				
No (#, %)	260 (67.0)	271 (68.1)	264 (69.3)	268 (67.7)
Light (#, %)	85 (21.9)	88 (22.1)	82 (21.5)	88 (22.2)
Moderate (#, %)	40 (10.3)	37 (9.3)	33 (8.7)	38 (9.6)
Heavy (#, %)	3 (0.8)	2 (0.5)	2 (0.5)	2 (0.5)
AM (95% CI)	2246.7 (1491.9, 3001.4)	2442.5 (948.3, 3936.7)	2205.5 (1179.9, 3231.2)	1952.0 (1238.8, 2665.2)
GM (95% CI)	12.1 (8.3, 17.4)	10.7 (7.5, 15.2)	9.5 (6.7, 13.5)	10.3 (7.3, 14.6)
*Trichuris trichiura*				
Prevalence (#, %)	100 (25.8)	83 (20.9)	68 (17.9)	103 (26.0)
Intensity				
No (#, %)	288 (74.2)	315 (79.1)	313 (82.2)	293 (74.0)
Light (#, %)	97 (25.0)	82 (20.6)	66 (17.3)	100 (25.3)
Moderate (#, %)	3 (0.8)	1 (0.3)	2 (0.5)	3 (0.7)
AM (95% CI)	57.5 (31.0, 84.0)	26.4 (16.8, 35.9)	34.1 (16.5, 51.8)	55.6 (36.8, 74.3)
GM (95% CI)	3.3 (2.7, 4.1)	2.5 (2.1, 2.9)	2.2 (1.9, 2.7)	3.4 (2.8, 4.2)
Hookworm				
Prevalence (#, %)	4 (1.0)	6 (1.5)	5 (1.3)	9 (2.3)
Intensity				
No (#, %)	384 (99.0)	392 (98.5)	376 (98.7)	387 (97.7)
Light (#, %)	4 (1.0)	6 (1.5)	5 (1.3)	9 (2.3)
AM (95% CI)	1.4 (-0.6, 3.4)	1.6 (-0.2, 3.3)	2.1 (-0.4, 4.7)	7.5 (-2.2, 17.2)
GM (95% CI)	1.0 (1.0, 1.1)	1.1 (1.0, 1.1)	1.1 (1.0, 1.1)	1.1 (1.0, 1.2)
Any STH infection				
Prevalence (#, %)	175 (45.1)	163 (41.0)	149 (39.1)	179 (45.2)

* STH results at all visits include only children whose specimens were analyzed by the Kato-Katz method (i.e. ^1^Group 1 and Group 3 at 12-month visit; ^2^Groups 2 and 3 at 18-month visit (results were not available for 74 children who were lost to follow-up); ^3^All groups at the 24-month visit (results were not available for 197 children who were lost to follow-up))

^ǂ1^Group 1 (MBD/PBO) = mebendazole at the 12-month visit and placebo at the 18-month visit; ^2^Group 2 (PBO/MBD) = placebo at the 12-month visit and mebendazole at the 18-month visit; ^3^Group 3 (MBD/MBD) = mebendazole at the 12 and 18-month visit; ^4^Group 4 (PBO/PBO) = placebo at the 12 and 18-month visit

^†^AM = arithmetic mean eggs per gram;

^§^GM = geometric mean eggs per gram. A value of 1 was added to each observation to calculate the geometric mean.

## References

[1] Joseph SA, Casapía M, Montresor A, Rahme E, Ward BJ, Marquis GS, et al (2015) The Effect of Deworming on Growth in One-Year-Old Children Living in a Soil-Transmitted Helminth-Endemic Area of Peru: A Randomized Controlled Trial. PLoS Negl Trop Dis 9(10): e0004020 doi: 10.1371/journal.pntd.0004020 26426270 10.1371/journal.pntd.0004020 PMC4591279

