# Supplementary material for: The Effect of Deworming on Growth in One-Year-Old Children Living in a Soil-Transmitted Helminth-Endemic Area of Peru: A Randomized Controlled Trial
Source: PLoS Negl Trop Dis. 2015 Oct 1;9(10):e0004020. doi: 10.1371/journal.pntd.0004020 (PMC4591279; doi:10.1371/journal.pntd.0004020)
Supplement: S2 Text — (PDF) [file pntd.0004020.s003.pdf]

IMPROVING EARLY CHILDHOOD GROWTH AND DEVELOPMENT IN RESOURCE-POOR LMICS BY INCORPORATING DEWORMING IN INTEGRATED CHILD HEALTH CARE

PRINCIPAL INVESTIGATOR
Dr. Theresa Gyorkos
Dept. of Epidemiology, Biostatistics, and Occupational Health - McGill University

CO-INVESTIGATORS
Dr. Martín Casapía
Asociación Civil Selva Amazónica – Iquitos, Peru
Ms. Serene Joseph
Dept. of Epidemiology, Biostatistics, and Occupational Health - McGill University
Dr. Grace Marquis
School of Nutrition and Dietetics – McGill University
Dr. Antonio Montresor
Neglected Tropical Diseases - World Health Organization
Dr. Brian Ward
Tropical Disease Centre – McGill University Health Centre

November 2010

TABLE OF CONTENTS

SUMMARY **3**

CONTEXT OF THE PRESENT PROTOCOL SUBMISSION **3**

LOCATION OF STUDY CONDUCT **4**

STUDY RATIONALE **4**

BACKGROUND **5**

- MALNUTRITION IN EARLY PRESCHOOL-AGE CHILDREN 5
- SOIL-TRANSMITTED HELMINTHS AND MALNUTRITION IN EARLY PRESCHOOL-AGE CHILDREN 5
- EVIDENCE ON DEWORMING IN 12 TO 24 MONTH OLD CHILDREN 6

OBJECTIVES **7**

METHODS **7**

- RESEARCH DESIGN 7
- STUDY COMMUNITY 8
- STUDY POPULATION 8
- SAMPLE SIZE 9
- RECRUITMENT 9
  - *Pre-recruitment* *9*
  - *Recruitment* *9*
- INTERVENTION GROUPS 10
- RANDOMIZATION AND TREATMENT ALLOCATION 10
- OUTCOME MEASUREMENTS 11
- LOSS TO FOLLOW-UP 12
- STANDARDIZATION AND QUALITY CONTROL 12
  - *Pre-recruitment training of field workers* *12*
  - *Quality control during fieldwork* *13*
- ANALYSES 13
  - *Primary analysis* *13*
  - *Secondary analyses* *13*

SAFETY ISSUES **14**

ANTICIPATED BENEFITS **14**

POTENTIAL RISKS **14**

PRIVACY AND CONFIDENTIALITY **14**

DETAILS OF THE RESEARCH TEAM **15**

ETHICAL OVERSIGHT **15**

COMPENSATION **15**

RESULT DISSEMINATION **16**

FUNDING **16**

TIMELINE OF RESEARCH ACTIVITIES **16**

REFERENCES **17**

ANNEX 1: INFORMED CONSENT .... ERROR! BOOKMARK NOT DEFINED.

Summary

Worldwide, over 2 billion people suffer from worm infections (hookworm, *Ascaris* and *Trichuris*, collectively referred to as soil-transmitted helminths (STHs)) in developing countries. STHs contribute to the overwhelming burden of poverty and deprivation in areas where adverse health, social, economic, education and other related factors predominate. STH infection in childhood results in short-term and lifelong disability, including malnutrition (e.g. underweight, stunting and wasting), cognitive impairment and increased susceptibility to other infection, among others. Mass deworming programs in school-age children are recommended by the World Health Organization (WHO). WHO also recommends deworming of preschool children (as of 12 months of age) in endemic areas; however, the benefits of deworming on improving growth and development, especially in the 12-24 month age group, have been inadequately studied. This knowledge is crucial because, with appropriate nutrition and health interventions, all children have a similar potential for healthy growth and development, provided that such interventions occur in the critical window of opportunity before the age of two.

Therefore, this double-blind randomized controlled trial will assess the benefit of deworming (mebendazole), integrated into routine child health care visits in a highly STH-endemic area (Iquitos, Peru), on the primary outcome of weight gain. Timing, frequency and impact of deworming will be considered. A total of 1760 children will be recruited at their routine 12-month check-up visit and randomly assigned to one of four intervention groups: Group 1 will receive usual care and mebendazole (single dose 500 mg) at their 12-month visit and usual care and a placebo tablet at their 18-month visit; Group 2 will receive usual care and a placebo tablet at their 12-month visit and usual care and mebendazole at their 18-month visit; Group 3 will receive usual care and mebendazole at both their 12-month and 18-month visit; and Group 4 will receive usual care at both their 12-month and 18-month visit. Usual care will consist of age-appropriate immunizations, supplements and other Peruvian Ministry of Health-recommended interventions. All children will be followed up to their 24-month visit and all will be given mebendazole at that time.

Additional secondary outcomes include length gain, motor and cognitive development and STH prevalence and intensity.

Improving child health is a priority area in global health research and a focus of the Millennium Development Goals. Early preschool-age children are at the most critical stage of growth and development and have been neglected in deworming programs. It is anticipated that the results will inform evidence-based policy on the provision of an integrated health package for young children in endemic areas and ultimately contribute to the reduction of health inequities in this vulnerable group.

Context of the Present Protocol Submission

The present proposal builds on past research conducted by all co-applicants in the fields of epidemiology, population health, intervention research, biostatistics, infection and nutrition, and especially, research conducted in Peru. This research has been interdisciplinary in nature, some of it participatory, some of it qualitative, and most, quantitative. The focus has been on malnutrition (eg. stunting, anemia) and the neglected tropical diseases of poverty, in particular, soil-transmitted helminth infections.

The current research question arises from both a global and a local evidence base on deworming. In 1996 and 1997, respectively, Gyorkos and Montresor conducted parasite surveys to inform the national deworming program in Guinée, West Africa [1,2]. This research contributed to national health policy, such that deworming activities at the district level were scaled up to national level within two years. With two other colleagues, in 2002, Gyorkos and Montresor co-authored a WHO manual on Helminth Control in School-Age Children which is a resource guide for managers of control programs [3]. (A second edition of this book is currently being prepared.) Both have been active in continuing deworming intervention research. Global initiatives, including that of WHO, now recognize the critical impact of deworming in pre-school children, in terms of its impact on growth and development.

Peru-based research began in 2001 with Gyorkos, Casapía and Rahme addressing the problem of anemia during pregnancy, half of which is generally attributable to worm infections. A double-blind randomized controlled trial, enrolling over 1000 pregnant women, was conducted to investigate the value of deworming and iron supplementation in reducing maternal anemia and improving infant birthweight [4-7]. The database from this research has provided the opportunity to address other research questions eg. [8]. Subsequent (published) research has included a survey of over 1000 schoolchildren living in Belén, a community of extreme poverty close to Iquitos, where we found that over one-third of Grade 5 students were stunted [9]; and by a household survey which documented underweight and wasting in ~ 30% of preschool children [10]. In the analysis of the pre-school-age child data, we also demonstrated the differences between the new WHO growth standards for pre-school children and the 'old' NCHS-CDC standards. Current research activities involving Gyorkos, Casapía, Joseph, and Rahme are partnered with the local Ministry of Health. They focus on evaluating the effectiveness of a set of health system-based nutrition educational interventions aimed at pregnant women and new mothers (to reduce infant and child malnutrition). Several manuscripts are already in preparation from this research[11-13]. In addition, our research now specifically includes gender analyses [14] and health equity considerations eg. [15].

LOCATION OF STUDY CONDUCT

The study will be conducted in neighbouring districts around the city of Iquitos, the capital of the Loreto region in the Peruvian Amazon. Highly STH-endemic areas in which the study will be conducted have been identified, including Belén, Punchana and San Juan. The principal investigator and several co-investigators have a long-standing research collaboration in these areas. The results of the study will be relevant to other STH-endemic areas throughout the world.

STUDY RATIONALE

Improving child health is a priority area in global health research and a focus of the Millennium Development Goals (MDGs). Early preschool-age children (i.e. less than two years of age) are at the most critical stage of growth and development and have been neglected in deworming intervention programs. The proposed study will determine the effectiveness of the timing and frequency of deworming on health and nutrition outcomes in children 12 to 24 months old, evidence which is currently lacking. The results will inform evidence-based policy on the incorporation of deworming activities into existing integrated health packages for young children, which currently include growth and development monitoring, vaccinations and

micronutrient supplementation. The trial will be conducted in a community of extreme poverty in a tropical area of Peru with results of relevance to other parts of the country, most of the LAC region, and STH-endemic areas of Africa and Asia. Scaling-up of interventions is needed in this age group to improve child nutrition on a global scale, to help achieve the MDGs, and ultimately to contribute to the reduction of health inequities in this vulnerable group [16,17]. These types of integrated, inter-sectoral prevention and control measures using existing health infrastructure are widely recognized as being essential to improve health and nutrition, reduce NTD burden and make progress towards the MDGs [18-21].

BACKGROUND

Malnutrition in early preschool-age children

Malnutrition is the leading cause of mortality in preschool-age children in low and middle income countries (LMICs), with over 150 million children suffering from one or more forms of malnutrition, including stunting, underweight and /or wasting [22,23]. Malnutrition also predisposes to infection, creating a vicious infection-malnutrition cycle that contributes to over 35% of the disease burden of early childhood [22]. Infection, including worm infections, and poor quality and low availability of food, resulting in micronutrient and other deficiencies, are the primary causes of malnutrition in childhood [24]. Early childhood is a critical time for growth faltering (related to weaning), and, as mobility increases, for early acquisition of infectious pathogens (through food, water and the environment). A recent study in 54 countries confirmed that interventions to prevent child malnutrition ***must occur during the first two years of life*** to prevent further growth deficits [17]. Interventions at this stage are essential to prevent both short- and longer-term adverse effects [25]. Stunting, in particular, is thought to be irreversible after 36 months of age [16]. Improving the health of the youngest children is integral to achieving the Millennium Development Goals (MDGs). Evidence from the World Health Organization (WHO) Child Growth Standards demonstrates that, with appropriate nutrition and health interventions, all children have a similar potential for healthy growth and development [26-28]; however, children living in areas of greatest poverty suffer the most from health and social inequities due to increased disease burden and lack of access to necessary health interventions and services [29].

Soil-transmitted helminths and malnutrition in early preschool-age children

Interventions that can help reduce child malnutrition within the first year of life include exclusive breastfeeding, micronutrient supplementation and vaccinations [16]. After one year of age, growth faltering often increases and children in developing countries continue to deviate even further from normal growth standards [17]. At this stage, it is critical to keep infection to a minimum and appropriate interventions should be incorporated into routine child health care packages [24]. In developing countries, the soil-transmitted helminths (STHs), or worm infections, are a significant contributor to poor health and malnutrition in all age groups. The STHs are a cluster of three diseases, *Ascariasis* (roundworm infection), *Trichuriasis* (whipworm infection) and hookworm infection. STHs are one of the most important Neglected Tropical Diseases (NTDs) and are the most common infections with over 2 billion people infected worldwide [30,31]. An estimated 5-10% are children in the most critical growth period under two years of age [30,31]. The burden of disease attributable to STH infections can be even more pronounced when children are exposed to these infections at an early age. As the STHs are spread through contaminated soil, food and hands, children begin to acquire these infections as they become mobile and begin to explore the environment [32]. *Ascaris* and *Trichuris* are the predominant STH

infections in early childhood [33].

The main adverse effect of STH infection in childhood is malnutrition. Other health consequences include cognitive impairment and increased susceptibility to infections, among others [34,35]. STH infection leads to malnutrition through nutrient malabsorption, blood loss and loss of appetite [34]. WHO recommends large-scale deworming interventions, using a single-dose anthelminthic (i.e. deworming) treatment, for the high-risk groups of school-age children, pregnant women and preschool-age children in endemic areas [36]. These interventions are often incorporated into schools (for school-age children) or prenatal care programs (for pregnant women). Children under two years of age had traditionally been excluded from deworming interventions as the burden of STH infection was perceived to be low in this age group. There has been increasing empirical evidence however, which shows that STH prevalence can be high in early childhood [37]. In a study in the Democratic Republic of the Congo, both *Ascaris* and *Trichuris* were prevalent in over 50% of children under two years of age [38]. In Belén, a community of extreme poverty in the Peruvian Amazon, the prevalence of *Ascaris* or *Trichuris* was only 4% in infants at 6 to 9 months of age, but rose to 30% at 12 to 14 months of age[39]. In this same area, the STH prevalence increased to 50% by 18 months of age (Gyorkos, unpublished data). It is becoming increasingly recognized that STH infection in early childhood may have important adverse effects as the parasites take up a greater proportion of the body in younger children [33]. WHO recommends including children 12 to 24 months of age in deworming activities in endemic areas [37], using either mebendazole (usual single-dose of 500 mg) or albendazole (reduced single-dose of 200 mg). (While some studies have included infants below 12 months of age in their study populations [40,41], WHO recommends that the minimum age for deworming interventions be 12 months.) Despite these recommendations, the benefit of deworming in this age group has been inadequately studied and coverage is still suboptimal. The global proportion of at-risk preschool-age children currently being dewormed is estimated to be on the order of 21%, with a low of 11% coverage in the Latin America and Caribbean (LAC) region [42].

Evidence on deworming in 12 to 24 month old children

A Cochrane review was published in 2008 looking at the effects of targeted deworming (i.e. targeted to high-risk groups of school-age and preschool-age children) on growth and school performance [43]. A total of 34 RCTs were included in the review; none focusing exclusively on the 12 to 24 month age group. Of the 34 trials, only two studies: 1) included children between 12 and 24 months of age in their child study populations; 2) were conducted in healthy children; 3) examined anthropometric outcomes (e.g. weight and/or height/length); and 4) compared either albendazole or mebendazole (the two drugs recommended by WHO for STH infection in children under two years of age [31]) to placebo or usual care [44,45]. Unfortunately, the child study populations in these two studies included children up to 7 years and 8 years of age, respectively, and reported results were not age-disaggregated; therefore, the effect of deworming in the 12-24 month age group is unknown. A recent meta-analysis was published by Hall et al [33], also in 2008, examining the effects of STH infection and deworming on growth and/or nutrition of children up to 18 years of age. This meta-analysis included 19 trials, the number being restricted to those conducted in areas where the prevalence of STH was over 50%. No trial reported specific data on 12-24 month-old children. One additional study, not included in either the Cochrane or the Hall reviews, found a significant benefit of albendazole on weight gain in children 1 to 5 years old, but, again, it was not possible to attribute this benefit to any particular age group [46].

Although these previous studies and reviews provide some evidence on the benefits of deworming on nutrition and health in children ***over*** two years of age, there are substantial limitations to extrapolating their results to children ***under*** two years of age. Of the previous studies, none have examined the effect exclusively in children 12 to 24 months or provided pertinent age-disaggregated data. With wide age ranges of children included in the sample size (up to 8 years of age), previous studies would have been underpowered to detect statistically significant effects of deworming in any particular age subgroup. No studies have determined the most appropriate time for deworming in children less than two years of age. Considering that STH prevalence is generally lower at 12 months of age (Gyorkos et al, manuscript under review), but can increase rapidly by 18 months of age (Gyorkos, unpublished data), this information is necessary to determine the time at which deworming can have maximum health and nutrition benefits. An additional limitation is that most studies used albendazole for the dewormed group; however, in the younger age groups who have more *Ascaris* and *Trichuris* infection, and negligible hookworm infection, deworming with mebendazole, rather than albendazole, would be more efficacious and produce a greater impact [47]. Lastly, as these younger children are undergoing rapid growth and development, appropriate indicators need to be measured to demonstrate the additional benefit that deworming may have, over and above the normal growth which takes place at this stage.

The Cochrane review, the meta-analysis and other publications have highlighted the limitations of previous studies (e.g. inadequate allocation concealment and blinding, high loss-to-follow-up, clustering of observations, inadequate data analyses, etc.) and recommended that better-designed RCTs are necessary to provide appropriate evidence on the benefits of deworming on multiple health and health-related outcomes [33,43,48]. In particular, evidence on the benefits for preschool-age children was deemed to be necessary for future intervention planning [33]. Considering the unique nutritional demands and growth patterns of younger children, aggregated results from older children do not provide an appropriate indication of the potential benefit of deworming on growth and nutrition in younger age groups who are in a critical growth window. Based on the limitations of previous studies, it is clear that methodologically sound research is needed to provide evidence on the benefit of incorporating deworming into routine health services for children between the ages of 12 and 24 months.

OBJECTIVES

The principal objective is to determine the effectiveness of a deworming intervention, in terms of timing, frequency and impact, to improve weight gain in infants between 12 and 24 months of age.

The secondary objectives are to determine the effectiveness of a deworming intervention, in terms of timing, frequency and impact, to improve length gain, cognitive and motor development and to reduce prevalence and intensity of STH infection in infants between 12 and 24 months of age.

METHODS

Research Design

The timing, frequency and impact of a deworming intervention, incorporated into health services during routine Growth and Development ('Crecimiento y Desarollo' or CREDE) visits, will be evaluated in children 12 to 24 months old living in a highly STH-endemic area of the Peruvian

Amazon using a double-blind randomized controlled trial design. There will be four intervention groups randomized to different deworming schedules: 1) usual care and deworming treatment at the 12-month visit and usual care and placebo at the 18-month visit, 2) usual care and placebo at the 12-month visit and usual care and deworming at the 18-month visit; 3) usual care and deworming at the 12-month visit and usual care and deworming at the 18-month visit; and 4) usual care and placebo at both the 12 and 18-month visits. All children will receive deworming at 24 months of age. Weight, length, cognitive and motor development and STH infection will be evaluated at 12, 18 and 24 months of age.

Study community
The study will be conducted in neighbouring districts around the city of Iquitos, the capital of the Loreto region in the Peruvian Amazon. Three districts of extreme poverty have been identified (Belén, Punchana and San Juan) where worm infections are highly endemic and malnutrition prevalence is high. The prevalence of underweight in children 12 to 14 months of age was recently estimated to be 31% and STH prevalence to be 29% (Gyorkos et al, manuscript under review). The STH prevalence doubles by 18 months of age (Gyorkos, unpublished data) and reaches over 85% in school-age children [9]. Previous research in the area has shown a significant association between STH infection and poor growth in both preschool- and school-age children [9,10] (Gyorkos et al, manuscript under review). Both STH infections and malnutrition have been identified as priority concerns by stakeholders in the community [49]. At present, there is no routine deworming for preschool-age children.

Preschool-age children attend routine government-sponsored CREDE visits at health clinics in Peru once-monthly from birth to 12 months of age, and every two months from 12 to 24 months of age (with less frequent visits continuing through preschool-age). Each health centre has one to two nurses who are responsible for CREDE check-ups. During routine CREDE visits, anthropometric measurements (e.g. weight and length) are taken, developmental milestones are recorded (e.g. gross motor skills), and children receive routine age-appropriate immunizations and supplements. Parents also receive nutrition and other health counselling for their child.

Study population
The study population will include children attending their routine 12-month CREDE visit (at which time measles vaccination is scheduled) in the study area. Using data provided by the Peruvian Ministry of Health, eight study health centres ("Centros de Salud") have been identified in high-risk (i.e. high STH prevalence) areas neighbouring Iquitos. These include Centros de Salud: 1) 06 de Octubre, 2) 09 de Octubre, 3) Belén, 4) Cardozo, 5) Bellavista de Nanay, 6) Masusa; 7) 01 de Enero and 8) Fernando Lores.

The inclusion and exclusion criteria for participating children are as follows:
Inclusion criteria: 1) children attending any one of the eight study health centres for their 12-month CREDE visit (for feasibility and to ensure recruitment of children from highly STH endemic areas); 2) children living in or near the Belén, Punchana or San Juan district (to ensure recruitment of children from highly STH endemic areas, as well as to ensure high follow-up)

Exclusion criteria: 1) children who are attending the clinic for suspected STH infection (as they may require immediate, specific treatment); 2) children who have received deworming treatment

in the six months prior to randomization (as the effect of previous treatment may interfere with the effect of the randomized treatment); 3) parents planning to move outside of the study area within the next 12 months (to maximize participation rates and follow-up); 4) children under 12 months of age (as treatment is not recommended for this age group), or 14 months of age or older (to ensure participants are at a comparable growth and developmental stage); and 5) children with serious congenital or chronic medical conditions and who would be considered by the attending staff not to benefit from deworming.

Sample size

Sample size calculations are based on the smallest meaningful difference in mean weight gain between intervention groups. From pilot data, we anticipate that the mean weight gain ± standard deviation between 12 and 24 months in the group receiving usual care and placebo at both time points will be approximately 2.0 kg ± 0.8 kg. Based on previous research, we expect an improvement of 0.5 kg in weight gain in the group receiving deworming at 18 months of age (with 50% STH prevalence) compared to the group receiving usual care and placebo at both 12 and 18-month visits (i.e. 2.5 kg) and 0.7 kg improvement in weight gain in the group receiving deworming at both 12 and 18 months of age (i.e. 2.7 kg) [51] . This difference takes into account effect dilution based on treating groups of both infected children (who will benefit directly from the deworming treatment) and non-infected children (who may not benefit directly from the deworming treatment). The mean weight gain in those receiving usual care and deworming at the 12-month visit is estimated to be half of the gain in the group receiving usual care and deworming at 18 months of age (i.e. 2.25 kg).

In order to have 80% power to detect a difference in mean weight gain of 0.2 kg (the smallest meaningful difference we are aiming to detect), assuming a common standard deviation of 0.8, and using a one-way ANOVA which accounts for pairwise multiple comparisons using the Tukey correction, the estimated sample size per group is 366 children. Taking into account a 20% attrition rate after 12 months of follow-up, the required sample size is estimated to be 1760 children, or 440 children per group (MCG4 Software, Ohio University, 2008).

Recruitment

Pre-recruitment

A pre-recruitment phase will occur at each participating health centre at which time mothers of children 9 to 11 months of age (who are not yet eligible to participate in the study) will be provided information on the study. They will be asked to return to the health centre on their child's first birthday at which time eligibility to participate in the study can be assessed. This step should facilitate recruitment, as well as help to ensure that children return to their health centre as close to their first birthday as possible.

Recruitment

During the recruitment phase of the study, eight project research nurses ('obstetriz') will each be present at one of the health centres during the morning CREDE clinics to recruit children into the study. The 'obstetriz' will obtain informed consent and administer the questionnaire to the parent/legal guardian, obtain the stool specimens for parasite assessment, obtain anthropometric measurements, assess cognitive and motor development, and administer the deworming (mebendazole) or placebo tablet.

Based on data provided by the eight identified health centres, approximately 200 to 250 children per month will be recruited for a total recruitment period of six months. Mothers routinely bring their children to the health centres in the mornings for check-ups and to receive their immunizations according to the Peruvian Ministry of Health schedule. A minimum response rate of 75-90% is expected based on previous experience working with this population [5,10].

Intervention groups
Children will be randomized into one of the following four intervention groups, following their baseline assessment at the 12-month visit. Treatment will be with a single-dose mebendazole tablet (500mg) or identical (in smell, taste and appearance) placebo and provided during their routine CREDE visit:

- **Group 1: Usual care and deworming at 12-month visit and usual care and placebo at 18-month visit.** Mebendazole will be given together with the standard health care package for 12-month-olds which includes administration of measles and rubella vaccine. Children in this group will not be dewormed at 18 months of age but will receive a placebo at this time along with the standard health care package for 18 month-olds which includes administration of the DPT (diphtheria, pertussis, and tetanus) vaccine booster.
- **Group 2: Usual care and placebo at 12-month visit and usual care and deworming at 18-month visit.** A placebo will be given together with the standard health care package for 12 month-olds (i.e., measles and rubella vaccine). Children in this group will receive mebendazole at 18 months of age along with the standard health care package for 18 month-olds which includes administration of the DPT vaccine booster.
- **Group 3: Usual care and deworming at both 12 and 18-month visits.** Mebendazole will be given together with the standard health care package for 12-month-olds which includes administration of measles and rubella vaccine. Children in this group will also receive mebendazole at the 18-month visit along with the standard health care package for 18 month-olds which includes administration of the DPT vaccine booster.
- **Group 4: Usual care and placebo at both 12 and 18-month visits.** A placebo will be given together with the standard health care package for 12-month-olds which includes administration of measles and rubella vaccine. Children in this group will also receive a placebo at the 18-month visit along with the standard health care package for 18 month-olds which includes administration of the DPT vaccine booster.

All children will receive deworming at the end of the one-year follow-up (ie. the 24-month visit).

No problems regarding compliance are expected. The deworming tablet is single-dose and it will be crushed and mixed with a fruit purée for ease of administration. This crushed tablet will be administered in the presence of the ‘obstetriz’.

Randomization and treatment allocation
Computer-generated randomly ordered blocks of eight and twelve will be used to randomly allocate children to each intervention group. This will ensure a balance in the number of children assigned to each group. Randomization will be stratified according to health centre. Sequentially numbered large envelopes will be prepared to meet the estimated sample size. Each large

envelope will contain the assigned intervention for each recruited child. Inside this envelope will be two smaller envelopes with the same ID number: one which will contain the intervention to be administered to the child at 12 months of age and one which will contain the intervention at 18 months of age. One pharmacist and one clinician-scientist not otherwise involved in the study will prepare envelopes containing the randomly chosen group intervention assignment in advance. Each 'obstetriz' will have a supply of 12-month envelopes containing the random intervention assignments at baseline. Once the single-dose mebendazole or placebo tablet is administered at the 12-month visit, the now empty 12-month intervention envelope will be stapled to each child's questionnaire (along with the signed informed consent form). This material will then be stored together at the research office with the corresponding 18-month envelope (with the same ID number) until the 18-month follow-up (at which time the tablet in the 18-month envelope will be administered to that child). Envelopes will be distributed among the recruitment sites until the sample size has been achieved.

Outcome measurements

Prior to randomization, a baseline assessment will be conducted which will include anthropometry, and cognitive and motor development. A questionnaire will be administered to the child's mother to obtain socio-demographic information, medical history, feeding practices, and environmental exposure history. Stool specimens will be collected from all children (a small amount of fruit may be given to children, as needed, to facilitate a bowel movement. This method has been used successfully in the past and does not interfere with the quality of the stool [9]). Follow-up assessments will be made at the 18-month CREDE visit, and at the 24-month CREDE visit, at which time all measurements will be repeated.

The primary outcome will be the mean weight gain between the 12 and the 24-month visit. Weight will be measured in duplicate using a portable electronic scale, accurate to the nearest 0.01 kg, calibrated daily using standard weights (Seca 334, Seca Corp., Baltimore, MD, USA).

Additional secondary measurements include length, cognitive and motor development and the prevalence and intensity of STH infections. Length (the recommended measurement of height in children < 30 months of age) will be measured in duplicate as recumbent crown-heel length on a flat surface using a measuring mat (Seca 210, Seca Corp., Baltimore, MD, USA), accurate to the nearest millimetre. Previous studies conducted by the proposed team in the Belén area showed inter and intra-rater reliability of over 95% for length/height and weight assessments (data not published), which are considered acceptable levels for anthropometric measurements [50,51]. Cognitive and motor development will be assessed using the Bayley scale for cognitive and motor development (Bayley Scales of Infant and Toddler Development, Third Edition. Pearson Education Inc, Texas).

Stool specimens will be collected from all children to assess the prevalence and intensity of STH infections. All stool specimens will be transferred to the laboratory at the local research office (the Asociación Civil Selva Amazónica); however, at the 12-month visit, only specimens from children receiving deworming treatment will be examined for the presence and intensity of STH infection (e.g. *Ascaris*, *Trichuris* and hookworm). At the 18-month CREDE visit, only stool specimens from children receiving deworming treatment will be analyzed. One member of the research team will be unblinded to treatment status to arrange for the appropriate specimens to be

analyzed. (All other research personnel will remain blinded to maintain the double-blinding of the trial). This approach to examining stool specimens from identified treatment groups is required so that children found infected will be treated, thus meeting the ethical requirement to treat children who are known to be infected (based on a positive stool specimen). This approach also ensures that a baseline (at 12 months) and interim (at 18 months) estimate of worm prevalence, and worm intensity (which requires a fresh stool specimen) is obtained. These specimens which are to be analyzed immediately will be examined by a trained microscopist using the Kato-Katz method. This is the recommended technique for assessment of the prevalence and intensity (eggs per gram) of intestinal parasitic infection in fresh stool [52]. For a one-stool specimen, sensitivity and specificity are over 96% for *Ascaris* and over 91% for *Trichuris* [53]. There is lower sensitivity and specificity for hookworm; however, hookworm infection prevalence is negligible in this age group, so this should not bias the results.

Stool specimens from children receiving usual care and placebo at 12 and/or 18 months will be preserved in sodium acetate-acetic acid-formalin (SAF) and analyzed upon completion of the study using the ether-concentration method, the recommended technique for estimating worm prevalence from preserved samples[54,55 54,55].

At the 24-month visit stool specimens from all four groups will be immediately analyzed using the Kato-Katz technique (as all will be receiving deworming). At this time, efficacy of the deworming treatment will also be assessed in a random sample of 100 infected children. Stool specimens for the efficacy study will be obtained on three consecutive days, 10 days after treatment.

Loss to follow-up

A 20% rate of attrition has been estimated after 12 months of follow-up. This estimate is based on over eight years of experience in the area which has included follow-up with pregnant women (less than 10 % loss to follow-up over 7 months) [5,10] and new mothers (less than 18 % loss to follow-up after 8 months)[13]. High participation rates and follow-up will be encouraged by providing reimbursement to cover travel costs (the equivalent of a roundtrip bus ticket) for each of the two follow-up visits (an incentive previously acceptable to both Canadian and Peruvian ethics committees). Mothers have a booklet that they bring to each routine CREDE visit so that essential information can be recorded and the following visit can be scheduled. This should also facilitate the scheduling of the follow-up visits. A higher attrition rate of 20% has been accounted for in the proposed study due to the longer follow-up time of 12 months.

Standardization and quality control

Pre-recruitment training of field workers

Prior to commencing recruitment, in-depth practical training of the research nurses ('obstetrices') will take place according to WHO guidelines [51,56]. An experienced anthropometrist from the Instituto de Investigación Nutricional (IIN) in Lima will conduct the training to ensure correct techniques for obtaining length and weight measurements. The anthropometrist will be designated as the "gold standard" to which observations from 'obstetrices' will be compared and standardized. 'Obstetrices' will work in pairs and record length and weight for 10 – 15 children between 12 and 24 months of age (who will not be part of the proposed trial). If the difference between 'obstetrices' is greater than 100g for weight or 7mm for length, measurements will be

re-taken to a maximum of three times. Precision will be assessed using the inter- and intra-rater technical error of measurement (TEM) and will be considered adequate if they are within the expert's 95% precision margin. An experienced social scientist will train 'obstetrices' on the administration of the Bayley scale for cognitive and motor development.

Quality control during fieldwork

Data collection activities during fieldwork will be regularly supervised to ensure that forms are completed correctly. All completed forms will be checked immediately after data collection at the end of each day. The consistency of egg count assessments will be evaluated among the laboratory technicians using standard quality control methods [52]. The laboratory supervisor will read 10% of the slides of each microscopist without prior knowledge of the result. In the case of a discrepancy larger than 10%, a discussion will take place between the laboratory supervisor and the microscopist to resolve the discrepancy and further slides will be examined to avoid repeated errors. The portable scales will be calibrated daily with standard weights, and the measuring mats will be calibrated daily using metal rods of specified lengths [51]. The IIN anthropometrist will conduct two additional training sessions during the study to ensure high validity and reliability of the anthropometric assessments throughout the follow-up period.

Analyses

Primary analysis

The results of the trial will be analyzed using an intention-to-treat approach. Following description of the study population at baseline, the mean weight gain between the first visit and the last follow-up (at the 24-month visit) will be compared between the four intervention groups using the one-way ANOVA procedure. While randomization should allow for approximately similar occurrence of determinants across the four intervention groups, adjustment for imbalances among the measured determinants and risk factors (e.g. history and duration of exclusive breastfeeding, timing of first introduction of complementary foods, home environment, family structure and characteristics and socioeconomic status) will be performed if needed using multivariate linear regression.

Secondary analyses

Secondary analyses will be conducted to examine differences between groups in terms of derived weight indices (i.e. mean weight-for-age Z score and proportion of underweight), length (mean length gain, length-for-age Z score and proportion of stunting), cognitive and motor development (mean score) and STH prevalence and intensity. Means will be compared using a one-way ANOVA procedure and proportions will be compared using chi-square statistics. Adjustment for imbalances among measured determinants and risk factors will be conducted as needed, using multivariate linear or logistic regression for continuous and dichotomous outcomes, respectively. Length-for-age and weight-for-age Z scores will be calculated using the WHO Anthro software (Version 3, 2009), which compares growth in the study population to a WHO standard population. In addition to age, this method also takes into account the sex of the child. Cut-offs used to categorize the continuous variables of weight, length and eggs per gram are based on WHO standards for underweight (<-2 SD), stunting (<-2 SD) and STH intensity (low and moderate/heavy, according to parasite-specific egg counts), respectively [36,52,57].

To evaluate the possible modification of baseline anthropometry on the effect of deworming, multivariate regression models (e.g. linear for continuous variables and logistic for dichotomous outcomes) will be used with baseline weight and length included as interaction terms. This analysis is included to document the added value of deworming among young children who are already faltering. It will provide insight into both the preventive nature of deworming and also into whether deworming can reverse growth faltering that has already occurred by 12 months of age. Finally, possible intra-cluster correlation (by health centre), will be investigated using the intraclass correlation coefficient. If clustering is indeed detected, hierarchical logistic regression models will be used to obtain accurate estimates of treatment effects.

SAFETY ISSUES

An expert panel of a WHO Informal Consultation in 2002 recommended that deworming, and specifically the usual dose of mebendazole (500mg), could be safely given to children 12 to 24 months of age [37]. In older age groups, side-effects are infrequent and, if present, are only mild and transient [33]. Therefore, negligible risks are anticipated to be associated with deworming in this study. There is some concern that deworming pills could pose a choking hazard to younger children [58]; however, the pills in this trial will be crushed and mixed with a fruit purée to eliminate this risk. The ascertainment of health and nutrition outcomes, including measurement of weight and length, carry no risk and will be conducted by the research team in a similar manner as is routinely conducted in early childhood health services.

ANTICIPATED BENEFITS

Currently, routine deworming of preschool-age children is not conducted in Peru. Children participating in the study will benefit directly by receiving deworming treatment at least once during the course of the study. Parents will also receive the laboratory results of the stool examinations upon completion of the study. The results of the study will inform health services in Peru and in other countries in providing deworming programs to preschool-age children.

POTENTIAL RISKS

There are no foreseeable risks associated with participation in this study. The deworming tablet, mebendazole, is very safe and effective and is being given to preschool-age children in many parts of the world. Most children do not have any side effects at all after deworming. If symptoms do occur, they are minor and temporary and usually disappear within 48 hours. PAHO and the World Health Organization consider mebendazole very safe and in fact recommend that it be given to preschool-age children where parasites are common.

PRIVACY AND CONFIDENTIALITY

Children will be assigned an identification number for the duration of the study to ensure the confidentiality of the results. All original documents, including questionnaires and informed consent forms, will be kept in a locked cabinet and room. Preserved stool specimens will be marked with the assigned identification number and kept in a locked refrigerator. All electronic information, such as databases, will be stored on a password-protected computer in a locked room. Access to original documents, electronic information and preserved stool specimens will be restricted to the Project Director, Principal Investigator and study coordinator only.

Details of the Research Team

Theresa Gyorkos is the Principal Investigator and will provide the overall leadership for all aspects of the trial. She will ensure that it is registered and that all Canadian and Peruvian ethics processes are complete and always current. She will be responsible for regular communication among the applicants, ensuring that all trial administrative and management issues are addressed appropriately and efficiently. Dr. Gyorkos will also supervise all academic and scientific aspects of the trial. **Martín Casapía** will provide local Peru-based supervision. Dr. Casapia is the local project director and will be accessible at all times to respond to urgent matters which may arise and which require immediate attention. He also provides key access to Ministry of Health officials and will be the local spokesperson for the project at an administrative level. **Antonio Montresor** brings to the team an extensive knowledge of field experience. He will provide input into all operational aspects of the fieldwork. In addition, he will provide an important liaison with WHO in understanding how the generated evidence will inform global health policy with respect to deworming in young children. **Grace Marquis** will also provide nutritional expertise on the project. She has conducted research on child nutrition in Peru and is now working in Ghana. She will be able to contextualize trial results to other pertinent geographic areas. She will provide insight into the biological processes underpinning the impact on young child growth and development from concurrent infection and malnutrition. **Elham Rahme** will provide biostatistical expertise and guidance during the data analysis. She has been extensively involved in pharmaco-epidemiological research and trials in Canada and will be able to provide methodological advice on RCT conduct. **Brian Ward** will provide expertise on the immunological processes at work during parasite infection in the malnourished host. **Serene Joseph**, a PhD Candidate in the Department of Epidemiology, Biostatistics and Occupational Health at McGill University, will be responsible for daily project management. Together with a full-time local research coordinator, she will be responsible for supervision of all field workers, for data entry and for overseeing quality control of data collection procedures (eg. questionnaire administration, laboratory examinations). Ms. Joseph will undertake all data analyses.

Ethical Oversight

The principal investigator, co-investigators and all other research personnel will conduct the study in an ethical manner which is consistent with the international principles of good research practice and will function according to institutional policies of the McGill University Health Centre and the Asociación Civil Selva Amazónica, governing human subject research and all applicable research guidelines. Ethics approval will be obtained and maintained from the Research Ethics Board in Canada and the National Institute of Health, the Ministry of Health, and the Instituto de Investigación Nutricional in Peru. The trial will be registered according to international regulations. The integrity of the trial with respect to blinding will be ensured in the following way: only one member of the research team will be unblinded to prepare the selected stool specimens for examination (i.e. that group that will be treated at either the 12 or 18-month time point). All other persons on the research team will remain blinded. Results of stool examinations will be available to mothers at the end of the study. Mothers of participating children will be properly informed of their rights as study subjects and informed consent will be obtained prior to randomization.

Compensation

There are no costs associated with participation in this study as visits will take place during

routine health centre visits according to the Peruvian Ministry of Health schedule. Study treatments and testing of stool specimens will be free of charge for the duration of the study. The cost of travel for the follow-up visits at the 18- and 24-month CREDE visit will be reimbursed to encourage participation and follow-up during the course of the study.

RESULT DISSEMINATION

The results of this trial will be published and presented in national and international fora, including scientific conferences and peer-reviewed journals. Presentations will also be made at the Pan American Health Organization (PAHO), WHO and in appropriate local, regional and national fora in Peru. The results will be of interest to all 130 STH-endemic countries that are currently, or that are planning to, implement deworming interventions in preschool-age populations. The results will be sent to newsletters such as Action Against Worms and to global initiatives such as Deworm the World, Children without Worms, the Mebendazole Advisory Committee, Partners for Parasite Control and Partnership for Child Development, among others.

FUNDING

This study is supported by a grant from the Thrasher Research Fund (Dr. Theresa W. Gyorkos). The duration of funding is three years, commencing on February 1, 2011.

TIMELINE OF RESEARCH ACTIVITIES

Table 1. Timeline of proposed activities for the randomized-controlled trial of deworming of 12 to 24 month old infants in the Peruvian Amazon.

	2010		2011				2012				2013	
**ACTIVITIES**	3rd Quarter	4th Quarter	1st Quarter	2nd Quarter	3rd Quarter	4th Quarter	1st Quarter	2nd Quarter	3rd Quarter	4th Quarter	1st Quarter	2nd Quarter
Ethics approval (Canada and Peru)	✓	✓	✓									
Purchase of study materials/supplies	✓	✓	✓									
Trial registration		✓										
Preliminary visits to health centres			✓									
Pre-testing of the questionnaire			✓									
Training of the research nurses			✓	✓								
Pre-recruitment period			✓	✓								
Recruitment period				✓	✓	✓						
18-month follow-up visit						✓	✓	✓				
24-month follow-up visit								✓	✓	✓		
Data analysis										✓	✓	
Reporting (presentations, manuscripts)										✓	✓	
Results dissemination												✓

References

1. Gyorkos TW, Camara B, Kokoskin E, Carabin H, Prouty R. Survey of parasitic prevalence in school-aged children in Guinea (1995) [in French]. Santé 1996;6:377-81.
2. Montresor A, Urbani C, Camara B, Bha B, Albonico M, Savioli L. Preliminary survey of implementation of a school health program in Guinea [in French]. Médecine Tropicale 1997;57:294-8.
3. WHO. Prevention and Control of Schistosomiasis and Soil-Transmitted Helminthiasis. Geneva, Switzerland: Report of a WHO Expert Committee, Technical Report Series, No 912 - World Health Organization; 2002.
4. Larocque R, Casapía M, Gotuzzo E, Gyorkos TW. Relationship between intensity of soil-transmitted helminth infections and anemia during pregnancy. American Journal of Tropical Medicine and Hygiene 2005;73:783-9.
5. Larocque R, Casapía M, Gotuzzo E, et al. A double-blind randomized controlled trial of antenatal mebendazole to reduce low birthweight in a hookworm-endemic area of Peru. Tropical Medicine & International Health 2006;11:1485-95.
6. Larocque R, Gyorkos TW. Should deworming be included in antenatal packages in hookworm-endemic areas of developing countries? Canadian Journal of Public Health Revue Canadienne de Sante Publique 2006;97:222-4.
7. Gyorkos TW, Larocque R, Casapía M, Gotuzzo E. Lack of risk of adverse birth outcomes after deworming in pregnant women. The Pediatric Infectious Disease Journal 2006;25:791-4.
8. Gilbert NL, Casapía M, Joseph SA, Ryan JA, Gyorkos TW. Inadequate prenatal care and the risk of stillbirth in the Peruvian Amazon. International Journal of Gynaecology and Obstetrics 2010;109:155-6.
9. Casapía M, Joseph SA, Núñez C, Rahme E, Gyorkos TW. Parasite risk factors for stunting in grade 5 students in a community of extreme poverty in Peru. International Journal for Parasitology 2006;36:741-7.
10. Casapía M, Joseph SA, Núñez C, Rahme E, Gyorkos TW. Parasite and maternal risk factors for malnutrition in preschool-age children in Belen, Peru using the new WHO Child Growth Standards. British Journal of Nutrition 2007;98:1259-6.
11. Blouin B, Casapía M, Aguilar E, et al. Delaying cord clamping: the effect of a two-component intervention to change practice in the Peruvian Amazon. BJOG : an international journal of obstetrics and gynaecology;Submitted.
12. Blouin B, Casapía M, Creed-Kanashiro H, et al. The effect of maternal anemia on the association between timing of umbilical cord clamping and infant anemia: observations of the hosptial practice in the Peruvian Amazon. Pediatrics 2010;Submitted.
13. Gyorkos TW, Maheu-Giroux M, Blouin B, et al. A hospital policy change towards delayed cord clamping is effective in improving hemoglobin levels and anemia status of eight month-old Peruvian infants. Bulletin of the World Health Organization 2010 Submitted.
14. Carsley S, Gyorkos TW. Sex and gender differences in hookworm infection in school-age children in Peru. in preparation.
15. Gyorkos TW, Joseph SA, Casapía M. Progress towards the Millennium Development Goals in a community of extreme poverty: local versus national disparities in Peru. Tropical Medicine & International Health 2009;14:645-52.
16. Bhutta Z, Ahmed T, Black RE, et al. What works? Interventions for maternal and child undernutrition and survival. The Lancet 2008;371:417-40.

17. Victora CG, de Onis M, Hallal PC, Blössner M, Shrimpton R. Worldwide Timing of Growth Faltering: Revisiting Implications for Interventions. Pediatrics 2010;125:473-80.
18. Ault SK. Pan American Health Organization's Regional Strategic Framework for adressing neglected diseases in neglected populations in Latin America and the Caribbean. Memórias do Instituto Oswaldo Cruz 2007;102:99-107.
19. Holveck JC, Ehrenberg JP, Ault SK, et al. Prevention, control, and elimination of neglected diseases in the Americas: pathways to integrated, inter-programmatic, inter-sectoral action for health and development. BMC Public Health 2007;7:6.
20. Hotez PJ, Bottazzi ME, Franco-Paredes C, Ault SK, Periago MR. The neglected tropical diseases of Latin America and the Caribbean: a review of disease burden and distribution and a roadmap for control and elimination. PLoS Neglected Tropical Diseases 2008;2:e300.
21. Disease Control Priorities Project. Deworming children brings huge health and development gains in low-income countries. Washington, DC: The World Bank; 2008.
22. Black RE, Allen LH, Bhutta ZA, et al. Maternal and child undernutrition: global and regional exposures and health consequences. The Lancet 2008;371:243-60.
23. de Onis M, Blössner M, Borghi E, Morris R, Frongillo EA. Methodology for estimating regional and global trends of child malnutrition. International Journal of Epidemiology 2004;33:1260-70.
24. Grantham-McGregor S, Cheung YB, Cueto S, et al. Developmental potential in the first 5 years for children in developing countries. The Lancet 2007;369:60-70.
25. Bryce J, Coitinho D, Darnton-Hill I, Pelletier D, Pinstrup-Andersen P, Maternal and Child Undernutrition Study Group. Maternal and child undernutrition: effective action at national level. The Lancet 2008;371:510-26.
26. de Onis M, Garza C, Onyango AW, Rolland-Cachera MF, le Comité de nutrition de la Société française de pédiatrie. WHO growth standards for infants and young children [in French]. Archives de Pédiatrie 2009;16:47-53.
27. Onyango AW. World Health Organization child growth standards: background, methodology and main results of the Multicentre Growth Reference Study [in French]. Archives de Pédiatrie 2009;16:735-6.
28. WHO Multicentre Growth Reference Study Group. Assessment of differences in linear growth among populations in the WHO Multicentre Growth Reference Study. Acta Paediatrica Supplementum 2006;450:56-65.
29. Belizán JM, Cafferata ML, Belizán M, Althabe F. Health inequality in Latin America. The Lancet 2007;370:1599-600.
30. de Silva NR, Brooker S, Hotez PJ, Montresor A, Engels D, Savioli L. Soil-transmitted helminth infections: updating the global picture. Trends in Parasitology 2003;19:547-51.
31. WHO. Report of the WHO informal consultation on the use of Praziquantel during pregnancy/lactation and Albendazole/Mebendazole in children under 24 months. Geneva, Switzerland: WHO/CDS/CPE/PVC; 2002.
32. Albonico M, Allen H, Chitsulo L, Engels D, Gabrielli A-F, Savioli L. Controlling soil-transmitted helminthiasis in pre-school-age children through preventive chemotherapy. PLoS Neglected Tropical Diseases 2008;2:e126.
33. Hall A, Hewitt G, Tuffrey V, de Silva N. A review and meta-analysis of the impact of intestinal worms on child growth and nutrition. Maternal and Child Nutrition 2008;4:118-236.
34. Crompton DW. The public health importance of hookworm diseases. Parasitology 2000;121:39-50.

35. O'Lorcain P, Holland CV. The public health importance of *Ascaris lumbricoides*. Parasitology 2000;121:51-71.
36. WHO. Deworming for health and development. Geneva, Switzerland: World Health Organization. WHO/CDS/CPE/PVC/2005.14; 2005.
37. Allen HE, Crompton DW, de Silva N, LoVerde PT, Olds GR. New policies for using anthelmintics in high risk groups. Trends in Parasitology 2002;18:381-2.
38. Mbendi M, Mashako MN, Lukuni M, Ndongola ZL. Albendzole in the treatment of intestinal nematodes in children aged 1-2 years old [in French]. Médicine Chirugie Digestive 1988;17:213-5.
39. Gyorkos TW, Maheu-Giroux M, Casapía M, Joseph SA, Creed-Kanashiro H. Stunting and early helminth infection in preschool-age children in the Amazon lowlands of Peru. Transactions of the Royal Society of Tropical Medicine and Hygiene 2010;In press.
40. Awasthi S, Pande VK. Six-monthly de-worming in infants to study effects on growth. Indian Journal of Pediatrics 2001;68:823-7.
41. Stoltzfus RJ, Chway HM, Montresor A, et al. Low dose daily iron supplementation improves iron status and appetite but not anemia, whereas quarterly anthelminthic treatment improves growth, appetite and anemia in Zanzibari preschool children. Journal of Nutrition 2004;134:348-56.
42. WHO. Soil-transmitted helminthiasis. Weekly epidemiological record 2008:237-52.
43. Taylor-Robinson DC, Jones AP, Garner P. Deworming drugs for treating soil-transmitted intestinal worms in children: effects on growth and school performance. Cochrane Database of Systematic Reviews 2007:CD000371.
44. Alderman H, Konde-Lule J, Sebuliba I, Bundy D, Hall A. Effect on weight gain of routinely giving albendazole to preschool children during child health days in Uganda: cluster randomised controlled trial. British Medical Journal 2006;333:122.
45. Kloetzel K, Merluzzi Filho TJ, Kloetzel D. Ascaris and malnutrition in a group of Brazilian children - a follow-up study. Journal of Tropical Pediatrics 1982;28:41-3.
46. Awasthi S, Peto R, Pande VK, Fletcher RH, Read S, Bundy DAP. Effects of deworming on malnourished preschool children in India: an open-labelled, cluster-randomized trial. PLoS Neglected Tropical Diseases 2008;2:e223.
47. Keiser J, Utzinger J. Efficacy of current drugs against soil-transmitted helminth infections. Systematic review and meta-analysis. Journal of the American Medical Association 2008;299:1937-48.
48. Bundy DA, Kremer M, Bleakley H, Jukes MC, Miguel E. Deworming and development: asking the right questions, asking the questions right. PLoS Neglected Tropical Diseases 2009;3:e362.
49. Casapía M, Joseph SA, Gyorkos TW. Multidisciplinary and participatory workshops with stakeholders in a community of extreme poverty in the Peruvian Amazon: Development of priority concerns and potential health, nutrition and education interventions. International Journal for Equity in Health 2007;6:6-14.
50. Ulijaszek SJ, Kerr DA. Anthropometric measurement error and the assessment of nutritional status. British Journal of Nutrition 1999;82:165-77.
51. de Onis M, Onyango AW, Van den Broeck J, Chumlea WC, Martorell R. Measurement and standardization protocols for anthropometry used in the construction of a new international growth reference. Food Nutr Bull 2004;25:S27-36.
52. Montresor A, Crompton DW, Bundy DAP, Hall A, Savioli L. Guidelines for the

evaluation of soil-transmitted helminthiasis and schistosomiasis at community level - A guide for managers of control programmes. Geneva, Switzerland: World Health Organization - WHO/CTD/SIP/98.1; 1998.
53. Tarafder MR, Carabin H, Joseph L, Balolong E, Jr., Olveda R, McGarvey ST. Estimating the sensitivity and specificity of Kato-Katz stool examination technique for detection of hookworms, *Ascaris lumbricoides* and *Trichuris trichiura* infections in humans in the absence of a 'gold standard'. International Journal for Parasitology 2010;40:399-404.
54. Glinz D, Silue KD, Knopp S, et al. Comparing diagnostic accuracy of Kato-Katz, Koga agar plate, ether-concentration, and FLOTAC for Schistosoma mansoni and soil-transmitted helminths. PLoS Negl Trop Dis 2010;4:e754.
55. Marti H, Escher E. [SAF--an alternative fixation solution for parasitological stool specimens]. Schweiz Med Wochenschr 1990;120:1473-6.
56. WHO Multicentre Growth Reference Study Group. Reliability of anthropometric measurements in the WHO Multicentre Growth Reference Study. Acta Paediatrica Supplement 2006;450:38-46.
57. WHO. WHO child growth standards: growth velocity based on weight, length and head circumference: methods and development. Geneva, Switzerland: Department of Nutrition for Health and Develpment. World Health Organization; 2009.
58. Albonico M, Shamlaye N, Shamlaye C, Savioli L. Control of intestinal parasitic infections in Seychelles: a comprehensive and sustainable approach. Bulletin of the World Health Organization 1996;74:577-86.
