# Supplementary material for: The Effect of Deworming on Growth in One-Year-Old Children Living in a Soil-Transmitted Helminth-Endemic Area of Peru: A Randomized Controlled Trial
Source: PLoS Negl Trop Dis. 2015 Oct 1;9(10):e0004020. doi: 10.1371/journal.pntd.0004020 (PMC4591279; doi:10.1371/journal.pntd.0004020)
Supplement: S1 Table — (DOCX) [file pntd.0004020.s004.docx]

**S1 Table**. Baseline characteristics of children who attended the 24-month visit (n=1563) compared to those who did not attend the 24-month visit (n=197).

|  | Attended 24-month visit | Did not attend 24-month visit |
| --- | --- | --- |
|  | (n=1563) | (n=197) |
| **Child characteristics** |  |  |
| Weight (kg) | 8.71(1.0) | 8.75 (1.0) |
| Length (cm) | 72.1 (2.4) | 72.4 (2.6) |
| Age (months) | 12.1 (0.3) | 12.2 (0.4) |
| Birth weight (kg) | 3.1 (0.5) | 3.1 (0.5) |
| Birth length (cm) | 49.4 (2.5) | 49.3 (2.4) |
| Sex (female) | 749 (47.9) | 91 (46.2) |
| Continued breastfeeding at 12 months | 1397 (89.4) | 178 (90.4) |
| Up-to-date vaccinations* | 1255 (80.5) | 155 (78.7) |
| Received vitamin A in previous year | 821 (52.5) | 100 (50.8) |
| Hospitalizations since birth | 148 (9.5) | 15 (7.6) |
| Walking without support | 387 (24.8) | 46 (23.5) |
| **Maternal characteristics** |  |  |
| Married or common-law | 1258 (80.5) | 165 (83.8) |
| Secondary education completed | 501 (32.1) | 53 (26.9) |
| Employment outside the home | 161 (10.3) | 18 (9.1) |
| **Household characteristics** |  |  |
| Periurban/rural residence | 1385 (88.6) | 175 (88.8) |
| Potable water in home | 801 (51.3) | 97 (49.2) |
| Earth or wood house material | 1205 (77.1) | 149 (75.6) |

Results are expressed as means (SD) or frequency (%)

*Up-to-date vaccinations include those scheduled between birth and 11 months of age (i.e. one dose of Bacille Calmette-Guérin (BCG), one dose of hepatitis B, three doses of polio, three doses of pentavalent, two doses of rotavirus, and two doses of pneumococcal)
