# Supplementary material for: The Effect of Deworming on Growth in One-Year-Old Children Living in a Soil-Transmitted Helminth-Endemic Area of Peru: A Randomized Controlled Trial
Source: PLoS Negl Trop Dis. 2015 Oct 1;9(10):e0004020. doi: 10.1371/journal.pntd.0004020 (PMC4591279; doi:10.1371/journal.pntd.0004020)
Supplement: S2 Table — (DOCX) [file pntd.0004020.s005.docx]

**S2 Table**. Effect of deworming on any STH and species-specific prevalence over 12 months, using a generalized linear model, complete case analysis* (n=1563).

|  | MBD/PBO**^1^ | PBO/MBD**^2^ | MBD/MBD**^3^ | PBO/PBO**^4^ |
| --- | --- | --- | --- | --- |
|  | (n=388) | (n=398) | (n=381) | (n=396) |
| **Outcome** |  |  |  |  |
| Any STH infection (yes vs. no) |  |  |  |  |
| Crude RR*** (95% CI) | 1.00 (0.86, 1.16) | 0.91 (0.77, 1.06) | 0.87 (0.73, 1.02) | reference |
| p-value | 0.98 | 0.23 | 0.09 |  |
| Adjusted RRǂ (95% CI) | 0.99 (0.85, 1.16) | 0.90 (0.77, 1.05) | 0.86 (0.73, 1.01) | reference |
| p-value | 0.92 | 0.19 | 0.07 |  |
|  |  |  |  |  |
| *Ascaris* infection (yes vs. no) |  |  |  |  |
| Crude RR (95% CI) | 1.02 (0.83, 1.25) | 0.99 (0.81, 1.21) | 0.95 (0.77, 1.17) | reference |
| p-value | 0.84 | 0.90 | 0.63 |  |
| Adjusted RR (95% CI) | 1.01 (0.83, 1.24) | 0.98 (0.80, 1.20) | 0.94 (0.77, 1.16) | reference |
| p-value | 0.89 | 0.83 | 0.59 |  |
|  |  |  |  |  |
| *Trichuris* infection (yes vs. no) |  |  |  |  |
| Crude RR (95% CI) | 0.99 (0.78, 1.26) | 0.80 (0.62, 1.03) | 0.69 (0.52, 0.90) | reference |
| p-value | 0.94 | 0.09 | 0.01 |  |
| Adjusted RR (95% CI) | 0.98 (0.77, 1.23) | 0.79 (0.61, 1.02) | 0.67 (0.51, 0.88) | reference |
| p-value | 0.84 | 0.07 | 0.01 |  |
|  |  |  |  |  |
| Hookworm infection (yes vs. no) |  |  |  |  |
| Crude RR (95% CI) | 0.45 (0.14, 1.46) | 0.66 (0.24, 1.85) | 0.58 (0.20, 1.71) | reference |
| p-value | 0.18 | 0.43 | 0.32 |  |
| Adjusted RR (95% CI) | 0.43 (0.14, 1.39) | 0.62 (0.22, 1.74) | 0.56 (0.19, 1.67) | reference |
| p-value | 0.16 | 0.36 | 0.30 |  |

* Complete case analysis includes data from children who attended the final 24-month trial visit

**^1^Group 1 (MBD/PBO) = mebendazole at the 12-month visit and placebo at the 18-month visit; ^2^Group 2 (PBO/MBD) = placebo at the 12-month visit and mebendazole at the 18-month visit; ^3^Group 3 (MBD/MBD) = mebendazole at the 12 and 18-month visit; ^4^Group 4 (PBO/PBO) = placebo at the 12 and 18-month visit

***RR = risk ratio

ǂ Adjusted models include age, sex and socioeconomic status
