# Supplementary material for: The Effect of Deworming on Growth in One-Year-Old Children Living in a Soil-Transmitted Helminth-Endemic Area of Peru: A Randomized Controlled Trial
Source: PLoS Negl Trop Dis. 2015 Oct 1;9(10):e0004020. doi: 10.1371/journal.pntd.0004020 (PMC4591279; doi:10.1371/journal.pntd.0004020)
Supplement: S3 Table — (DOCX) [file pntd.0004020.s006.docx]

**S3 Table**. Overall effect of deworming on anthropometric outcomes over 12 months, using one-way ANOVA and multivariable linear regression analysis, per-protocol analysis* (n=1103).

|  | MBD/PBO**^1^ | PBO/MBD**^2^ | MBD/MBD**^3^ | PBO/PBO**^4^ |
| --- | --- | --- | --- | --- |
|  | (n=280) | (n=281) | (n=275) | (n=267) |
| **Outcome** |  |  |  |  |
| Weight gain, kg | 2.08 | 1.90 | 2.06 | 2.02 |
| (95% CI) | (2.00, 2.16) | (1.81, 1.99) | (1.98, 2.15) | (1.94, 2.11) |
| Unadjusted difference | 0.06 | -0.13 | 0.04 | reference |
| (95% CI) | (-0.07, 0.18) | (-0.25, 0.00) | (-0.08, 0.16) |  |
| p-value | 0.372 | 0.042 | 0.538 |  |
| Adjusted differenceǂ | 0.06 | -0.11 | 0.04 | reference |
| (95% CI) | (-0.07, 0.18) | (-0.24, 0.01) | (-0.08, 0.16) |  |
| p-value | 0.371 | 0.065 | 0.497 |  |
|  |  |  |  |  |
| Length gain, cm | 9.90 | 9.55 | 9.75 | 9.68 |
| (95% CI) | (9.66, 10.14) | (9.32, 9.77) | (9.53, 9.98) | (9.45, 9.92) |
| Unadjusted difference | 0.22 | -0.14 | 0.07 | reference |
| (95% CI) | (-0.11, 0.54) | (-0.46, 0.19) | (-0.26, 0.40) |  |
| p-value | 0.192 | 0.410 | 0.667 |  |
| Adjusted difference | 0.26 | -0.07 | 0.12 | reference |
| (95% CI) | (-0.06, 0.58) | (-0.39, 0.25) | (-0.20, 0.45) |  |
| p-value | 0.107 | 0.690 | 0.445 |  |
|  |  |  |  |  |
| WAZ†^1^ change | -0.18 | -0.38 | -0.21 | -0.25 |
| (95% CI) | (-0.26, -0.10) | (-0.47, -0.30) | (-0.29, -0.14) | (-0.33, -0.18) |
| Unadjusted difference | 0.07 | -0.13 | 0.04 | reference |
| (95% CI) | (-0.04, 0.18) | (-0.24, -0.19) | (-0.07, 0.15) |  |
| p-value | 0.213 | 0.021 | 0.466 |  |
| Adjusted difference | 0.06 | -0.11 | 0.05 | reference |
| (95% CI) | (-0.04, 0.18) | (-0.22, -0.01) | (-0.06, 0.16) |  |
| p-value | 0.218 | 0.040 | 0.411 |  |
|  |  |  |  |  |
| LAZ†^2^ change | -0.46 | -0.62 | -0.52 | -0.55 |
| (95% CI) | (-0.54, -0.38) | (-0.70, -0.55) | (-0.59, -0.44) | (-0.63, -0.47) |
| Unadjusted difference | 0.10 | -0.07 | 0.04 | reference |
| (95% CI) | (-0.02, 0.21) | (-0.18, 0.04) | (-0.07, 0.15) |  |
| p-value | 0.092 | 0.206 | 0.522 |  |
| Adjusted difference | 0.11 | -0.04 | 0.06 | reference |
| (95% CI) | (0.00, 0.22) | (-0.15, 0.07) | (-0.05, 0.17) |  |
| p-value | 0.052 | 0.439 | 0.310 |  |

Results are expressed as mean (95% Confidence Interval)

* Per-protocol analysis includes data from children who attended all three study visits and did not report receiving deworming outside of the trial protocol

**^1^Group 1 (MBD/PBO) = mebendazole at the 12-month visit and placebo at the 18-month visit; ^2^Group 2 (PBO/MBD) = placebo at the 12-month visit and mebendazole at the 18-month visit; ^3^Group 3 (MBD/MBD) = mebendazole at the 12 and 18-month visit; ^4^Group 4 (PBO/PBO) = placebo at the 12 and 18-month visit

ǂ Adjusted models include age, sex, socioeconomic status and continued breastfeeding at 12 months of age

†^1^WAZ=weight-for-age z score; ^2^LAZ=length-for-age z score. Z scores were derived using WHO international growth standards [36]
