# Supplementary material for: The Effect of Deworming on Growth in One-Year-Old Children Living in a Soil-Transmitted Helminth-Endemic Area of Peru: A Randomized Controlled Trial
Source: PLoS Negl Trop Dis. 2015 Oct 1;9(10):e0004020. doi: 10.1371/journal.pntd.0004020 (PMC4591279; doi:10.1371/journal.pntd.0004020)
Supplement: S4 Table — (DOCX) [file pntd.0004020.s007.docx]

**S4 Table.** Overall effect of deworming on anthropometric outcomes over 12 months, using one-way ANOVA and multivariable linear regression analysis, complete case analysis* (n=1563).

|  | MBD/PBO**^1^ | PBO/MBD**^2^ | MBD/MBD**^3^ | PBO/PBO**^4^ |
| --- | --- | --- | --- | --- |
|  | (n=388) | (n=398) | (n=381) | (n=396) |
| **Outcome** |  |  |  |  |
| Weight gain, kg | 2.05 | 1.94 | 2.04 | 2.00 |
| (95% CI) | (1.98, 2.12) | (1.86, 2.02) | (1.97, 2.12) | (1.94, 2.07) |
| Unadjusted difference | 0.05 | -0.06 | 0.04 | reference |
| (95% CI) | (-0.05, 0.15) | (-0.17, 0.04) | (-0.06, 0.15) |  |
| p-value | 0.336 | 0.214 | 0.418 |  |
| Adjusted differenceǂ | 0.05 | -0.06 | 0.05 | reference |
| (95% CI) | (-0.05, 0.16) | (-0.16, 0.05) | (-0.06, 0.15) |  |
| p-value | 0.296 | 0.278 | 0.371 |  |
|  |  |  |  |  |
| Length gain, cm | 9.84 | 9.57 | 9.69 | 9.64 |
| (95% CI) | (9.64, 10.05) | (9.38, 9.75) | (9.50, 9.87) | (9.45, 9.84) |
| Unadjusted difference | 0.20 | -0.07 | 0.04 | reference |
| (95% CI) | (-0.07, 0.47) | (-0.35, 0.20) | (-0.23, 0.32) |  |
| p-value | 0.151 | 0.589 | 0.753 |  |
| Adjusted difference | 0.22 | -0.06 | 0.08 | reference |
| (95% CI) | (-0.05, 0.49) | (-0.32, 0.21) | (-0.19, 0.35) |  |
| p-value | 0.104 | 0.673 | 0.562 |  |
|  |  |  |  |  |
| WAZ†^1^ change | -0.23 | -0.36 | -0.23 | -0.28 |
| (95% CI) | (-0.30, -0.16) | (-0.43, -0.29) | (-0.30, -0.17) | (-0.34, -0.22) |
| Unadjusted difference | 0.05 | -0.08 | 0.05 | reference |
| (95% CI) | (-0.05, 0.14) | (-0.17, 0.01) | (-0.05, 0.14) |  |
| p-value | 0.322 | 0.086 | 0.328 |  |
| Adjusted difference | 0.05 | -0.07 | 0.05 | reference |
| (95% CI) | (-0.04, 0.14) | (-0.16, 0.02) | (-0.04, 0.14) |  |
| p-value | 0.256 | 0.145 | 0.290 |  |
|  |  |  |  |  |
| LAZ†^2^ change | -0.51 | -0.63 | -0.55 | -0.57 |
| (95% CI) | (-0.58, -0.44) | (-0.69, -0.56) | (-0.61, -0.49) | (-0.64, -0.51) |
| Unadjusted difference | 0.06 | -0.05 | 0.03 | reference |
| (95% CI) | (-0.03, 0.15) | (-0.14, 0.04) | (-0.07, 0.12) |  |
| p-value | 0.192 | 0.261 | 0.585 |  |
| Adjusted difference | 0.07 | -0.04 | 0.04 | reference |
| (95% CI) | (-0.02, 0.17) | (-0.13, 0.05) | (-0.05, 0.13) |  |
| p-value | 0.107 | 0.405 | 0.412 |  |

Results are expressed as mean (95% Confidence Interval)

* Complete case analysis includes data from children who attended the final 24-month trial visit

**^1^Group 1 (MBD/PBO) = mebendazole at the 12-month visit and placebo at the 18-month visit; ^2^Group 2 (PBO/MBD) = placebo at the 12-month visit and mebendazole at the 18-month visit; ^3^Group 3 (MBD/MBD) = mebendazole at the 12 and 18-month visit; ^4^Group 4 (PBO/PBO) = placebo at the 12 and 18-month visit

ǂ Adjusted models include age, sex, socioeconomic status and continued breastfeeding at 12 months of age

†^1^WAZ=weight-for-age z score; ^2^LAZ=length-for-age z score. Z scores were derived using WHO international growth standards [[36](#_ENREF_34)]
