# Supplementary material for: The Effect of Deworming on Growth in One-Year-Old Children Living in a Soil-Transmitted Helminth-Endemic Area of Peru: A Randomized Controlled Trial
Source: PLoS Negl Trop Dis. 2015 Oct 1;9(10):e0004020. doi: 10.1371/journal.pntd.0004020 (PMC4591279; doi:10.1371/journal.pntd.0004020)
Supplement: S5 Table — (DOCX) [file pntd.0004020.s008.docx]

**S5 Table**. Overall effect of deworming on anthropometric outcomes over 12 months, using one-way ANOVA and multivariable linear regression analysis, restricted to STH-infected children at baseline* (n=186).

|  | MBD/PBO**^1^ | PBO/MBD**^2^ | MBD/MBD**^3^ | PBO/PBO**^4^ |
| --- | --- | --- | --- | --- |
|  | (n=50) | (n=48) | (n=56) | (n=32) |
| **Outcome** |  |  |  |  |
| Weight gain, kg | 1.91 | 2.04 | 2.03 | 2.07 |
| (95% CI) | (1.71, 2.11) | (1.84, 2.24) | (1.84, 2.22) | (1.86, 2.27) |
| Unadjusted difference | -0.16 | -0.03 | -0.04 | reference |
| (95% CI) | (-0.46, 0.15) | (-0.33, 0.28) | (-0.34, 0.26) |  |
| p-value | 0.313 | 0.870 | 0.791 |  |
| Adjusted differenceǂ | -0.20 | -0.06 | -0.09 | reference |
| (95% CI) | (-0.52, 0.11) | (-0.37, 0.25) | (-0.39, 0.22) |  |
| p-value | 0.199 | 0.708 | 0.571 |  |
|  |  |  |  |  |
| Length gain, cm | 9.20 | 9.55 | 9.36 | 9.88 |
| (95% CI) | (8.78, 9.61) | (9.06, 10.04) | (8.89, 9.82) | (9.27, 10.49) |
| Unadjusted difference | -0.68 | -0.33 | -0.52 | reference |
| (95% CI) | (-1.42, 0.05) | (-1.07, 0.41) | (-1.24, 0.20) |  |
| p-value | 0.068 | 0.382 | 0.154 |  |
| Adjusted difference | -0.68 | -0.33 | -0.48 | reference |
| (95% CI) | (-1.42, 0.07) | (-1.08, 0.41) | (-1.21, 0.25) |  |
| p-value | 0.077 | 0.376 | 0.193 |  |
|  |  |  |  |  |
| WAZ†^1^ change | -0.35 | -0.22 | -0.20 | -0.19 |
| (95% CI) | (-0.54, -0.17) | (-0.41, -0.03) | (-0.38, -0.01) | (-0.39, 0.01) |
| Unadjusted difference | -0.17 | -0.03 | -0.01 | reference |
| (95% CI) | (-0.46, 0.12) | (-0.32, 0.27) | (-0.29, 0.28) |  |
| p-value | 0.261 | 0.853 | 0.957 |  |
| Adjusted difference | -0.23 | -0.08 | -0.07 | reference |
| (95% CI) | (-0.53, 0.06) | (-0.37, 0.22) | (-0.36, 0.21) |  |
| p-value | 0.125 | 0.615 | 0.612 |  |
|  |  |  |  |  |
| LAZ†^2^ change | -0.71 | -0.58 | -0.60 | -0.46 |
| (95% CI) | (-0.85, -0.57) | (-0.76, -0.40) | (-0.77, -0.43) | (-0.67, -0.24) |
| Unadjusted difference | -0.25 | -0.12 | -0.14 | reference |
| (95% CI) | (-0.52, 0.01) | (-0.39, 0.14) | (-0.40, 0.11) |  |
| p-value | 0.058 | 0.362 | 0.277 |  |
| Adjusted difference | -0.26 | -0.14 | -0.14 | reference |
| (95% CI) | (-0.52, 0.01) | (-0.40, 0.12) | (-0.40, 0.12) |  |
| p-value | 0.059 | 0.299 | 0.276 |  |

Results are expressed as mean (95% Confidence Interval)

* Subgroup analysis includes data from children who were positive for STH infection at baseline by the direct method (Groups 2 and 4) or by the Kato-Katz method (Groups 1 and 3)

**^1^Group 1 (MBD/PBO) = mebendazole at the 12-month visit and placebo at the 18-month visit; ^2^Group 2 (PBO/MBD) = placebo at the 12-month visit and mebendazole at the 18-month visit; ^3^Group 3 (MBD/MBD) = mebendazole at the 12 and 18-month visit; ^4^Group 4 (PBO/PBO) = placebo at the 12 and 18-month visit

ǂ Adjusted models include age, sex, socioeconomic status and continued breastfeeding at 12 months of age

†^1^WAZ=weight-for-age z score; ^2^LAZ=length-for-age z score. Z scores were derived using WHO international growth standards [[36](#_ENREF_34)]
