# Supplementary material for: The Effect of Deworming on Growth in One-Year-Old Children Living in a Soil-Transmitted Helminth-Endemic Area of Peru: A Randomized Controlled Trial
Source: PLoS Negl Trop Dis. 2015 Oct 1;9(10):e0004020. doi: 10.1371/journal.pntd.0004020 (PMC4591279; doi:10.1371/journal.pntd.0004020)
Supplement: S6 Table — (DOCX) [file pntd.0004020.s009.docx]

S6 Table. The effect of timing of deworming on anthropometric outcomes over 12 months, using one-way ANOVA and multivariable linear regression analyses, intention-to-treat analysis (n=880*).

|  | MBD/PBO**^1^ | PBO/MBD**^2^ |
| --- | --- | --- |
|  | (n=440) | (n=440) |
| **Primary outcome** |  |  |
| Weight gain, kg | 2.05 | 1.93 |
| (95% CI) | (1.98, 2.13) | (1.85, 2.02) |
| Unadjusted difference | 0.12 | reference |
| (95% CI) | **(0.01, 0.23)** |  |
| p-value | 0.033 |  |
| Adjustedǂ difference | 0.12 | reference |
| (95% CI) | **(0.01, 0.23)** |  |
| p-value | 0.035 |  |
|  |  |  |
| **Secondary outcomes** |  |  |
| Length gain, cm | 9.84 | 9.53 |
| (95% CI) | (9.64, 10.05) | (9.33, 9.74) |
| Unadjusted difference | 0.31 | reference |
| (95% CI) | **(0.04, 0.58)** |  |
| p-value | 0.026 |  |
| Adjusted difference | 0.31 | reference |
| (95% CI) | **(0.05, 0.58)** |  |
| p-value | 0.021 |  |
|  |  |  |
| WAZ†^1^ change | -0.23 | -0.36 |
| (95% CI) | (-0.30, -0.16) | (-0.43, -0.29) |
| Unadjusted difference | 0.13 | reference |
| (95% CI) | **(0.03, 0.23)** |  |
| p-value | 0.009 |  |
| Adjusted difference | 0.12 | reference |
| (95% CI) | **(0.03, 0.22)** |  |
| p-value | 0.011 |  |
|  |  |  |
| LAZ†^2^ change | -0.51 | -0.64 |
| (95% CI) | (-0.58, -0.44) | (-0.71, -0.57) |
| Unadjusted difference | 0.12 | reference |
| (95% CI) | **(0.03, 0.21)** |  |
| p-value | 0.007 |  |
| Adjusted difference | 0.12 | reference |
| (95% CI) | **(0.03, 0.21)** |  |
| p-value | 0.007 |  |

Results are expressed as mean (95% Confidence Interval)

* Intention-to-treat analysis includes data from 1563 children for whom final outcome information was available, and 197 children who were lost to follow-up and whose outcome information was estimated using multiple imputation

**^1^Group 1 (MBD/PBO) = mebendazole at the 12-month visit and placebo at the 18-month visit; ^2^Group 2 (PBO/MBD) = placebo at the 12-month visit and mebendazole at the 18-month visit

ǂ Adjusted models include age, sex, socioeconomic status and continued breastfeeding at 12 months of age

†^1^WAZ=weight-for-age z score; ^2^LAZ=length-for-age z score. Z scores were derived using WHO international growth standards [36]
