# Supplementary material for: The Effect of Deworming on Growth in One-Year-Old Children Living in a Soil-Transmitted Helminth-Endemic Area of Peru: A Randomized Controlled Trial
Source: PLoS Negl Trop Dis. 2015 Oct 1;9(10):e0004020. doi: 10.1371/journal.pntd.0004020 (PMC4591279; doi:10.1371/journal.pntd.0004020)
Supplement: S7 Table — (DOCX) [file pntd.0004020.s010.docx]

**S7 Table**. The effect of timing of deworming on anthropometric outcomes over 12 months, using one-way ANOVA and multivariable linear regression analysis, per-protocol analysis* (n=561).

|  | MBD/PBO**^1^ | PBO/MBD**^2^ |
| --- | --- | --- |
|  | (n=280) | (n=281) |
| **Outcome** |  |  |
| Weight gain, kg | 2.08 | 1.90 |
| (95% CI) | (2.00, 2.16) | (1.81, 1.99) |
| Unadjusted difference | 0.18 | reference |
| (95% CI) | (0.06, 0.30) |  |
| p-value | 0.003 |  |
| Adjusted differenceǂ | 0.17 | reference |
| (95% CI) | (0.05, 0.29) |  |
| p-value | 0.006 |  |
|  |  |  |
| Length gain, cm | 9.90 | 9.55 |
| (95% CI) | (9.66, 10.14) | (9.32, 9.77) |
| Unadjusted difference | 0.35 | reference |
| (95% CI) | (0.03, 0.68) |  |
| p-value | 0.031 |  |
| Adjusted difference | 0.33 | reference |
| (95% CI) | (0.01, 0.64) |  |
| p-value | 0.042 |  |
|  |  |  |
| WAZ†^1^ change | -0.18 | -0.38 |
| (95% CI) | (-0.26, -0.10) | (-0.47, -0.30) |
| Unadjusted difference | 0.20 | reference |
| (95% CI) | (0.09, 0.31) |  |
| p-value | 0.0003 |  |
| Adjusted difference | 0.18 | reference |
| (95% CI) | (0.08, 0.29) |  |
| p-value | 0.001 |  |
|  |  |  |
| LAZ†^2^ change | -0.46 | -0.62 |
| (95% CI) | (-0.54, -0.38) | (-0.70, -0.55) |
| Unadjusted difference | 0.17 | reference |
| (95% CI) | (0.06, 0.28) |  |
| p-value | 0.003 |  |
| Adjusted difference | 0.15 | reference |
| (95% CI) | (0.04, 0.26) |  |
| p-value | 0.006 |  |

Results are expressed as mean (95% Confidence Interval)

* Per-protocol analysis includes data from children who attended all three study visits and did not report receiving deworming outside of the trial protocol

**^1^Group 1 (MBD/PBO) = mebendazole at the 12-month visit and placebo at the 18-month visit; ^2^Group 2 (PBO/MBD) = placebo at the 12-month visit and mebendazole at the 18-month visit

ǂ Adjusted models include age, sex, socioeconomic status and continued breastfeeding at 12 months of age

†^1^WAZ=weight-for-age z score; ^2^LAZ=length-for-age z score. Z scores were derived using WHO international growth standards [36]
