# Supplementary material for: The Effect of Deworming on Growth in One-Year-Old Children Living in a Soil-Transmitted Helminth-Endemic Area of Peru: A Randomized Controlled Trial
Source: PLoS Negl Trop Dis. 2015 Oct 1;9(10):e0004020. doi: 10.1371/journal.pntd.0004020 (PMC4591279; doi:10.1371/journal.pntd.0004020)
Supplement: S8 Table — (DOCX) [file pntd.0004020.s011.docx]

**S8 Table**. The effect of timing of deworming on anthropometric outcomes over 12 months, using one-way ANOVA and multivariable linear regression analysis, complete case analysis* (n=786).

|  | MBD/PBO**^1^ | PBO/MBD**^2^ |
| --- | --- | --- |
|  | (n=388) | (n=398) |
| **Outcome** |  |  |
| Weight gain, kg | 2.05 | 1.94 |
| (95% CI) | (1.98, 2.12) | (1.86, 2.02) |
| Unadjusted difference | 0.12 | reference |
| (95% CI) | (0.01, 0.22) |  |
| p-value | 0.028 |  |
| Adjusted differenceǂ | 0.12 | reference |
| (95% CI) | (0.01, 0.21) |  |
| p-value | 0.033 |  |
|  |  |  |
| Length gain, cm | 9.84 | 9.57 |
| (95% CI) | (9.64, 10.05) | (9.38, 9.75) |
| Unadjusted difference | 0.27 | reference |
| (95% CI) | (0.00, 0.55) |  |
| p-value | 0.048 |  |
| Adjusted difference | 0.28 | reference |
| (95% CI) | (0.01, 0.55) |  |
| p-value | 0.041 |  |
|  |  |  |
| WAZ†^1^ change | -0.23 | -0.36 |
| (95% CI) | (-0.30, -0.16) | (-0.43, -0.29) |
| Unadjusted difference | 0.13 | reference |
| (95% CI) | (0.03, 0.22) |  |
| p-value | 0.007 |  |
| Adjusted difference | 0.12 | reference |
| (95% CI) | (0.03, 0.21) |  |
| p-value | 0.010 |  |
|  |  |  |
| LAZ†^2^ change | -0.51 | -0.63 |
| (95% CI) | (-0.58, -0.44) | (-0.69, -0.56) |
| Unadjusted difference | 0.11 | reference |
| (95% CI) | (0.02, 0.21) |  |
| p-value | 0.015 |  |
| Adjusted difference | 0.11 | reference |
| (95% CI) | (0.02, 0.20) |  |
| p-value | 0.015 |  |

Results are expressed as mean (95% Confidence Interval)

* Complete case analysis includes data from children who attended the final 24-month trial visit

**^1^Group 1 (MBD/PBO) = mebendazole at the 12-month visit and placebo at the 18-month visit; ^2^Group 2 (PBO/MBD) = placebo at the 12-month visit and mebendazole at the 18-month visit

ǂ Adjusted models include age, sex, socioeconomic status and continued breastfeeding at 12 months of age

†^1^WAZ=weight-for-age z score; ^2^LAZ=length-for-age z score. Z scores were derived using WHO international growth standards [36]
