# Supplementary material for: The Effect of Deworming on Growth in One-Year-Old Children Living in a Soil-Transmitted Helminth-Endemic Area of Peru: A Randomized Controlled Trial
Source: PLoS Negl Trop Dis. 2015 Oct 1;9(10):e0004020. doi: 10.1371/journal.pntd.0004020 (PMC4591279; doi:10.1371/journal.pntd.0004020)
Supplement: S9 Table — (DOCX) [file pntd.0004020.s012.docx]

**S9 Table.** The effect of timing of deworming on anthropometric outcomes over 12 months, using one-way ANOVA and multivariable linear regression analysis, restricted to STH-infected children at baseline* (n=98).

|  | MBD/PBO**^1^ | PBO/MBD**^2^ |
| --- | --- | --- |
|  | (n=50) | (n=48) |
| **Outcome** |  |  |
| Weight gain, kg | 1.91 | 2.04 |
| (95% CI) | (1.71, 2.11) | (1.84, 2.24) |
| Unadjusted difference | -0.13 | reference |
| (95% CI) | (-0.40, 0.14) |  |
| p-value | 0.344 |  |
| Adjusted differenceǂ | -0.15 | reference |
| (95% CI) | (-0.42, 0.13) |  |
| p-value | 0.298 |  |
|  |  |  |
| Length gain, cm | 9.20 | 9.55 |
| (95% CI) | (8.78, 9.61) | (9.06, 10.04) |
| Unadjusted difference | -0.35 | reference |
| (95% CI) | (-1.01, 0.30) |  |
| p-value | 0.287 |  |
| Adjusted difference | -0.34 | reference |
| (95% CI) | (-1.00, 0.32) |  |
| p-value | 0.308 |  |
|  |  |  |
| WAZ†^1^ change | -0.35 | -0.22 |
| (95% CI) | (-0.54, -0.17) | (-0.41, -0.03) |
| Unadjusted difference | -0.14 | reference |
| (95% CI) | (-0.40, 0.12) |  |
| p-value | 0.293 |  |
| Adjusted difference | -0.16 | reference |
| (95% CI) | (-0.42, 0.10) |  |
| p-value | 0.238 |  |
|  |  |  |
| LAZ†^2^ change | -0.71 | -0.58 |
| (95% CI) | (-0.85, -0.57) | (-0.76, -0.40) |
| Unadjusted difference | -0.13 | reference |
| (95% CI) | (-0.37, 0.10) |  |
| p-value | 0.270 |  |
| Adjusted difference | -0.11 | reference |
| (95% CI) | (-0.35, 0.12) |  |
| p-value | 0.326 |  |

Results are expressed as mean (95% Confidence Interval)

* Subgroup analysis includes data from children who were positive for STH infection at baseline by the direct method (MBD/PBO) or by the Kato-Katz method (PBO/MBD)

**^1^Group 1 (MBD/PBO) = mebendazole at the 12-month visit and placebo at the 18-month visit; ^2^Group 2 (PBO/MBD) = placebo at the 12-month visit and mebendazole at the 18-month visit

ǂ Adjusted models include age, sex, socioeconomic status and continued breastfeeding at 12 months of age

†^1^WAZ=weight-for-age z score; ^2^LAZ=length-for-age z score. Z scores were derived using WHO international growth standards [36]
