# Supplementary material for: The Effect of Deworming on Growth in One-Year-Old Children Living in a Soil-Transmitted Helminth-Endemic Area of Peru: A Randomized Controlled Trial
Source: PLoS Negl Trop Dis. 2015 Oct 1;9(10):e0004020. doi: 10.1371/journal.pntd.0004020 (PMC4591279; doi:10.1371/journal.pntd.0004020)
Supplement: S10 Table — (DOCX) [file pntd.0004020.s013.docx]

S10 Table. The effect of frequency of deworming on anthropometric outcomes over 12 months, using one-way ANOVA and multivariable linear regression analyses, intention-to-treat analysis (n=1320*).

|  | MBD/PBO**^1^ | PBO/MBD**^2^ | MBD/MBD**^3^ |
| --- | --- | --- | --- |
|  | (n=440) | (n=440) | (n=440) |
| **Primary outcome** |  |  |  |
| Weight gain, kg | 2.05 | 1.93 | 2.04 |
| (95% CI) | (1.98, 2.13) | (1.85, 2.02) | (1.97, 2.11) |
| Unadjusted difference | 0.02 | -0.10 | reference |
| (95% CI) | (-0.09, 0.12) | **(-0.20, -0.01)** |  |
| p-value | 0.777 | 0.039 |  |
| Adjustedǂ difference | 0.01 | -0.11 | reference |
| (95% CI) | (-0.10, 0.12) | **(-0.21, -0.01)** |  |
| p-value | 0.891 | 0.029 |  |
|  |  |  |  |
| **Secondary outcomes** |  |  |  |
| Length gain, cm | 9.84 | 9.53 | 9.67 |
| (95% CI) | (9.64, 10.05) | (9.33, 9.74) | (9.50, 9.85) |
| Unadjusted difference | 0.17 | -0.14 | reference |
| (95% CI) | (-0.10, 0.44) | (-0.41, 0.13) |  |
| p-value | 0.219 | 0.313 |  |
| Adjusted difference | 0.14 | -0.18 | reference |
| (95% CI) | (-0.13, 0.40) | (-0.44, 0.09) |  |
| p-value | 0.309 | 0.199 |  |
|  |  |  |  |
| WAZ†^1^ change | -0.23 | -0.36 | -0.24 |
| (95% CI) | (-0.30, -0.16) | (-0.43, -0.29) | (-0.30, -0.18) |
| Unadjusted difference | 0.01 | -0.12 | reference |
| (95% CI) | (-0.09, 0.10) | **(-0.21, -0.03)** |  |
| p-value | 0.892 | 0.007 |  |
| Adjusted difference | 0.01 | -0.12 | reference |
| (95% CI) | (-0.09, 0.11) | **(-0.20, -0.03)** |  |
| p-value | 0.849 | 0.010 |  |
|  |  |  |  |
| LAZ†^2^ change | -0.51 | -0.64 | -0.56 |
| (95% CI) | (-0.58, -0.44) | (-0.71, -0.57) | (-0.62, -0.49) |
| Unadjusted difference | 0.04 | -0.08 | reference |
| (95% CI) | (-0.05, 0.13) | (-0.18, 0.01) |  |
| p-value | 0.383 | 0.072 |  |
| Adjusted difference | 0.04 | -0.08 | reference |
| (95% CI) | (-0.05, 0.13) | (-0.17, 0.01) |  |
| p-value | 0.337 | 0.086 |  |

Results are expressed as mean (95% Confidence Interval).

* Intention-to-treat analysis includes data from 1563 children for whom final outcome information was available, and 197 children who were lost to follow-up and whose outcome information was estimated using multiple imputation.

**^1^Group 1 (MBD/PBO) = mebendazole at the 12-month visit and placebo at the 18-month visit; ^2^Group 2 (PBO/MBD) = placebo at the 12-month visit and mebendazole at the 18-month visit; ^3^Group 3 (MBD/MBD) = mebendazole at the 12 and 18-month visit.

ǂ Adjusted models include age, sex, socioeconomic status and continued breastfeeding at 12 months of age

†^1^WAZ=weight-for-age z score; ^2^LAZ=length-for-age z score. Z scores were derived using WHO international growth standards [36]
