# Supplementary material for: The Effect of Deworming on Growth in One-Year-Old Children Living in a Soil-Transmitted Helminth-Endemic Area of Peru: A Randomized Controlled Trial
Source: PLoS Negl Trop Dis. 2015 Oct 1;9(10):e0004020. doi: 10.1371/journal.pntd.0004020 (PMC4591279; doi:10.1371/journal.pntd.0004020)
Supplement: S11 Table — (DOCX) [file pntd.0004020.s014.docx]

**S11 Table**. The effect of frequency of deworming on anthropometric outcomes over 12 months, using one-way ANOVA and multivariable linear regression analysis, per-protocol analysis* (n=836).

|  | MBD/PBO**^1^ | PBO/MBD**^2^ | MBD/MBD**^3^ |
| --- | --- | --- | --- |
|  | (n=280) | (n=281) | (n=275) |
| **Outcome** |  |  |  |
| Weight gain, kg | 2.08 | 1.90 | 2.06 |
| (95% CI) | (2.00, 2.16) | (1.81, 1.99) | (1.98, 2.15) |
| Unadjusted difference | 0.02 | -0.16 | reference |
| (95% CI) | (-0.10, 0.14) | (-0.29, -0.04) |  |
| p-value | 0.782 | 0.008 |  |
| Adjusted differenceǂ | 0.01 | -0.16 | reference |
| (95% CI) | (-0.11, 0.13) | (-0.28, -0.04) |  |
| p-value | 0.832 | 0.011 |  |
|  |  |  |  |
| Length gain, cm | 9.90 | 9.55 | 9.75 |
| (95% CI) | (9.66, 10.14) | (9.32, 9.77) | (9.53, 9.98) |
| Unadjusted difference | 0.15 | -0.21 | reference |
| (95% CI) | (-0.18, 0.47) | (-0.53, 0.11) |  |
| p-value | 0.379 | 0.206 |  |
| Adjusted difference | 0.14 | -0.19 | reference |
| (95% CI) | (-0.18, 0.45) | (-0.51, 0.13) |  |
| p-value | 0.396 | 0.239 |  |
|  |  |  |  |
| WAZ†^1^ change | -0.18 | -0.38 | -0.21 |
| (95% CI) | (-0.26, -0.10) | (-0.47, -0.30) | (-0.29, -0.14) |
| Unadjusted difference | 0.03 | -0.17 | reference |
| (95% CI) | (-0.08, 0.14) | (-0.28, -0.06) |  |
| p-value | 0.606 | 0.002 |  |
| Adjusted difference | 0.02 | -0.16 | reference |
| (95% CI) | (-0.09, 0.13) | (-0.27, -0.05) |  |
| p-value | 0.683 | 0.004 |  |
|  |  |  |  |
| LAZ†^2^ change | -0.46 | -0.62 | -0.52 |
| (95% CI) | (-0.54, -0.38) | (-0.70, -0.55) | (-0.59, -0.44) |
| Unadjusted difference | 0.06 | -0.11 | reference |
| (95% CI) | (-0.05, 0.17) | (-0.22, 0.00) |  |
| p-value | 0.295 | 0.055 |  |
| Adjusted difference | 0.05 | -0.10 | reference |
| (95% CI) | (-0.06, 0.16) | (-0.21, 0.01) |  |
| p-value | 0.353 | 0.070 |  |

Results are expressed as mean (95% Confidence Interval)

* Per-protocol analysis includes data from children who attended all three study visits and did not report receiving deworming outside of the trial protocol

**^1^Group 1 (MBD/PBO) = mebendazole at the 12-month visit and placebo at the 18-month visit; ^2^Group 2 (PBO/MBD) = placebo at the 12-month visit and mebendazole at the 18-month visit; ^3^Group 3 (MBD/MBD) = mebendazole at the 12 and 18-month visit

ǂ Adjusted models include age, sex, socioeconomic status and continued breastfeeding at 12 months of age

†^1^WAZ=weight-for-age z score; ^2^LAZ=length-for-age z score. Z scores were derived using WHO international growth standards [36]
