# Supplementary material for: The Effect of Deworming on Growth in One-Year-Old Children Living in a Soil-Transmitted Helminth-Endemic Area of Peru: A Randomized Controlled Trial
Source: PLoS Negl Trop Dis. 2015 Oct 1;9(10):e0004020. doi: 10.1371/journal.pntd.0004020 (PMC4591279; doi:10.1371/journal.pntd.0004020)
Supplement: S12 Table — (DOCX) [file pntd.0004020.s015.docx]

**S12 Table**. The effect of frequency of deworming on anthropometric outcomes over 12 months, using one-way ANOVA and multivariable linear regression analysis, complete case analysis* (n=1167).

|  | MBD/PBO**^1^ | PBO/MBD**^2^ | MBD/MBD**^3^ |
| --- | --- | --- | --- |
|  | (n=388) | (n=398) | (n=381) |
| **Outcome** |  |  |  |
| Weight gain, kg | 2.05 | 1.94 | 2.04 |
| (95% CI) | (1.98, 2.12) | (1.86, 2.02) | (1.97, 2.12) |
| Unadjusted difference | 0.01 | -0.11 | reference |
| (95% CI) | (-0.10, 0.11) | (-0.21, 0.00) |  |
| p-value | 0.883 | 0.041 |  |
| Adjusted differenceǂ | 0.01 | -0.10 | reference |
| (95% CI) | (-0.10, 0.11) | (-0.21, 0.00) |  |
| p-value | 0.884 | 0.049 |  |
|  |  |  |  |
| Length gain, cm | 9.84 | 9.57 | 9.69 |
| (95% CI) | (9.64, 10.05) | (9.38, 9.75) | (9.50, 9.87) |
| Unadjusted difference | 0.16 | -0.12 | reference |
| (95% CI) | (-0.12, 0.43) | (-0.39, 0.16) |  |
| p-value | 0.267 | 0.395 |  |
| Adjusted difference | 0.14 | -0.14 | reference |
| (95% CI) | (-0.13, 0.41) | (-0.41, 0.13) |  |
| p-value | 0.302 | 0.318 |  |
|  |  |  |  |
| WAZ†^1^ change | -0.23 | -0.36 | -0.23 |
| (95% CI) | (-0.30, -0.16) | (-0.43, -0.29) | (-0.30, -0.17) |
| Unadjusted difference | 0.00 | -0.13 | reference |
| (95% CI) | (-0.09, 0.09) | (-0.22, -0.03) |  |
| p-value | 0.994 | 0.007 |  |
| Adjusted difference | 0.00 | -0.12 | reference |
| (95% CI) | (-0.09, 0.10) | (-0.21, -0.03) |  |
| p-value | 0.943 | 0.012 |  |
|  |  |  |  |
| LAZ†^2^ change | -0.51 | -0.63 | -0.55 |
| (95% CI) | (-0.58, -0.44) | (-0.69, -0.56) | (-0.61, -0.49) |
| Unadjusted difference | 0.04 | -0.08 | reference |
| (95% CI) | (-0.06, 0.13) | (-0.17, 0.01) |  |
| p-value | 0.453 | 0.097 |  |
| Adjusted difference | 0.04 | -0.08 | reference |
| (95% CI) | (-0.06, 0.13) | (-0.17, 0.01) |  |
| p-value | 0.436 | 0.100 |  |

Results are expressed as mean (95% Confidence Interval)

* Complete case analysis includes data from children who attended the final 24-month trial visit

**^1^Group 1 (MBD/PBO) = mebendazole at the 12-month visit and placebo at the 18-month visit; ^2^Group 2 (PBO/MBD) = placebo at the 12-month visit and mebendazole at the 18-month visit; ^3^Group 3 (MBD/MBD) = mebendazole at the 12 and 18-month visit

ǂ Adjusted models include age, sex, socioeconomic status and continued breastfeeding at 12 months of age

†^1^WAZ=weight-for-age z score; ^2^LAZ=length-for-age z score. Z scores were derived using WHO international growth standards [36]
