# Supplementary material for: The Effect of Deworming on Growth in One-Year-Old Children Living in a Soil-Transmitted Helminth-Endemic Area of Peru: A Randomized Controlled Trial
Source: PLoS Negl Trop Dis. 2015 Oct 1;9(10):e0004020. doi: 10.1371/journal.pntd.0004020 (PMC4591279; doi:10.1371/journal.pntd.0004020)
Supplement: S13 Table — (DOCX) [file pntd.0004020.s016.docx]

**S13 Table**. The effect of frequency of deworming on anthropometric outcomes over 12 months, using one-way ANOVA and multivariable linear regression analysis, restricted to STH-infected children at baseline* (n=154).

|  | MBD/PBO**^1^ | PBO/MBD**^2^ | MBD/MBD**^3^ |
| --- | --- | --- | --- |
|  | (n=50) | (n=48) | (n=56) |
| **Outcome** |  |  |  |
| Weight gain, kg | 1.91 | 2.04 | 2.03 |
| (95% CI) | (1.71, 2.11) | (1.84, 2.24) | (1.84, 2.22) |
| Unadjusted difference | -0.12 | 0.01 | reference |
| (95% CI) | (-0.38, 0.15) | (-0.25, 0.28) |  |
| p-value | 0.383 | 0.913 |  |
| Adjusted differenceǂ | -0.12 | 0.03 | reference |
| (95% CI) | (-0.38, 0.15) | (-0.24, 0.30) |  |
| p-value | 0.381 | 0.832 |  |
|  |  |  |  |
| Length gain, cm | 9.20 | 9.55 | 9.36 |
| (95% CI) | (8.78, 9.61) | (9.06, 10.04) | (8.89, 9.82) |
| Unadjusted difference | -0.16 | 0.19 | reference |
| (95% CI) | (-0.79, 0.47) | (-0.44, 0.83) |  |
| p-value | 0.615 | 0.551 |  |
| Adjusted difference | -0.19 | 0.15 | reference |
| (95% CI) | (-0.82, 0.44) | (-0.49, 0.79) |  |
| p-value | 0.549 | 0.644 |  |
|  |  |  |  |
| WAZ†^1^ change | -0.35 | -0.22 | -0.20 |
| (95% CI) | (-0.54, -0.17) | (-0.41, -0.03) | (-0.38, -0.01) |
| Unadjusted difference | -0.16 | -0.02 | reference |
| (95% CI) | (-0.41, 0.09) | (-0.27, 0.23) |  |
| p-value | 0.213 | 0.878 |  |
| Adjusted difference | -0.16 | 0.00 | reference |
| (95% CI) | (-0.40, 0.09) | (-0.25, 0.25) |  |
| p-value | 0.214 | 0.995 |  |
|  |  |  |  |
| LAZ†^2^ change | -0.71 | -0.58 | -0.60 |
| (95% CI) | (-0.85, -0.57) | (-0.76, -0.40) | (-0.77, -0.43) |
| Unadjusted difference | -0.11 | 0.02 | reference |
| (95% CI) | (-0.34, 0.11) | (-0.21, 0.25) |  |
| p-value | 0.330 | 0.865 |  |
| Adjusted difference | -0.11 | 0.00 | reference |
| (95% CI) | (-0.34, 0.11) | (-0.22, 0.23) |  |
| p-value | 0.320 | 0.972 |  |

Results are expressed as mean (95% Confidence Interval)

* Subgroup analysis includes data from children who were positive for STH infection at baseline by the direct method (PBO/MBD) or by the Kato-Katz method (MBD/PBO and MBD/MBD)

**^1^Group 1 (MBD/PBO) = mebendazole at the 12-month visit and placebo at the 18-month visit; ^2^Group 2 (PBO/MBD) = placebo at the 12-month visit and mebendazole at the 18-month visit; ^3^Group 3 (MBD/MBD) = mebendazole at the 12 and 18-month visit

ǂ Adjusted models include age, sex, socioeconomic status and continued breastfeeding at 12 months of age

†^1^WAZ=weight-for-age z score; ^2^LAZ=length-for-age z score. Z scores were derived using WHO international growth standards [36]
