# Supplementary material for: The Effect of Deworming on Growth in One-Year-Old Children Living in a Soil-Transmitted Helminth-Endemic Area of Peru: A Randomized Controlled Trial
Source: PLoS Negl Trop Dis. 2015 Oct 1;9(10):e0004020. doi: 10.1371/journal.pntd.0004020 (PMC4591279; doi:10.1371/journal.pntd.0004020)
Supplement: S1 Text — (PDF) [file pntd.0004020.s002.pdf]

L'Hôpital de Montréal pour enfants
The Montreal Children's Hospital

Centre universitaire de santé McGill
McGill University Health Centre

March 7, 2011

Dr. Theresa Gyorkos
Clinical Epidemiology
MUHC – Royal Victoria Hospital
V building

Re: 10-242-PED **Improving Early Childhood Growth and Development in Resource-poor LMICs by Incorporating Deworming in Integrated Child Health Care**
Funded by: **Thrasher Research Fund**

Dear Dr. Gyorkos,

The above-named research proposal received Full Board review at the convened meeting of the Montreal Children's Hospital Research Ethics Board on December 13, 2010 and was found to be within ethical guidelines for conduct at the McGill University Health Centre, and was entered accordingly into the minutes of the Research Ethics Board (REB) meeting. At the MUHC, sponsored research activities that require US federal assurance are conducted under Federal Wide Assurance (FWA) 00000840.

We are pleased to inform you that final approval was provided on March 4, 2011 for the following:

- MUHC Initial Review form & Annexe B
- MCH science approval letter (dated 12 Dec. 2010)
- Thrasher Research Fund reviewer comments & approval letter dated October 25, 2010
- Protocol, version November 2010
- Consent, English version 2 (revised) Feb. 15, 2011

All research involving human subjects requires review at a recurring interval and the current study approval is in effect until March 3, 2012 (anniversary of initial approval). It is the responsibility of the principal investigator to submit an Application for Continuing Review to the REB prior to the expiration of approval to comply with the regulation for continuing review of "at least once per year".

The Research Ethics Boards (REBs) of the McGill University Health Centre are registered REBs working under the published guidelines of the Tri-Council Policy Statement, in compliance with the "Plan d'action ministériel en éthique de la recherche et en intégrité scientifique" (MSSS, 1998) and the Food and Drugs Act (7 June, 2001), acting in conformity with standards set forth in the (US) Code of Federal Regulations governing human subjects research, and functioning in a manner consistent with internationally accepted principles of good clinical practice.

We wish to advise you that this document completely satisfies the requirement for Research Ethics Board Attestation as stipulated by Health Canada.

The project was assigned MUHC Study Number **10-242-PED** that is required as MUHC reference when communicating about the research. Should any revision to the study, or other unanticipated development occur prior to the next required review, you must advise the REB without delay. Regulation does not permit initiation of a proposed study modification prior to REB approval for the amendment.

Sincerely,

Jane McDonald, M.D., F.R.C.P.C
Chairperson, Research Ethics Board

L'HÔPITAL DE MONTRÉAL POUR ENFANTS • THE MONTREAL CHILDREN'S HOSPITAL
2300, rue Tupper, Montréal (Québec) Canada H3H 1P3 Tél. : 514 412-4400
www.hopitalpourenfants.com www.thechildren.com

CONSTANCIA

El que suscribe, Presidente del Comité Institucional de Ética (CIE) de la Universidad Peruana Cayetano Heredia certifica que ha evaluado el Proyecto de Investigación titulado: **"Mejorar el crecimiento y desarrollo en la infancia temprana en países de bajos recursos a través de un programa de desparasitación incorporado en la atención integrada de salud infantil."**, versión 2.0 de fecha 06 de julio del 2011, código de inscripción **11005** presentado por el Investigador Principal, **Dr. Martín Casapía**

Considerando que:

1. El protocolo de investigación mantiene los lineamientos **científicos y éticos**, con balance favorable entre **riesgos y beneficios.**
2. El equipo investigador se encuentra calificado para su ejecución.
3. El Consentimiento Informado, versión 4.0 de fecha 06 de Julio del 2011 brinda la información suficiente de manera adecuada y permite el enrolamiento voluntario de los participantes.
4. Están contemplados mecanismos de **confidencialidad** de los datos clínicos

De acuerdo a los estándares propuestos por la Universidad.

Por lo tanto, el CIE acuerda **EXPEDIR LA APROBACION**, en fecha **09 de Agosto del 2011** por seguir las Guías de Buenas Prácticas Clínicas.

Cualquier **enmienda** al protocolo original, así como la ocurrencia de alguna **eventualidad** que afecte adversamente a los participantes, deberá ser reportada en breve plazo a este Comité.

La **cobertura de gastos**, en caso de lesiones debidas al estudio, será proporcionada por el Organismo Patrocinador.

El Centro de Investigación deberá informar y detallar en forma escrita los motivos de una suspensión y cancelación del Ensayo Clínico.

..../////

Av. Honorio Delgado 430, lima 31, Perú. Apartado Postal 4314, Lima 100
Teléfono: (511) 319-0000 Anexo: 2271 / 2542 **Telefax:** 482-4541
E-mail: duict@oficinas-upch.pe
Página Web: http://www.upch.edu.pe/vrinve/duict/

////....

El Centro de Investigación remitirá al Comité una copia fedateada de la aprobación expedida por la Oficina General de Investigación y Transferencia Tecnológica del Instituto Nacional de Salud.

El investigador reportará cada 6 meses el progreso del estudio y alcanzará un **informe** al término de éste.

Esta aprobación tendrá **vigencia hasta el 08 de Agosto del 2012**. Si aplica, los trámites para su renovación deberán iniciarse por lo menos 30 días previos a su vencimiento.

El investigador presentará un informe al término del estudio.

Lima, 09 de Agosto de 2011

Dr. Leonid Lecca García
Presidente (e)
Comité Institucional de Ética
/zrmg

COMITÉ INSTITUCIONAL DE ÉTICA UNIVERSIDAD PERUANA CAYETANO HEREDIA DUICT

Av. Honorio Delgado 430, lima 31, Perú. Apartado Postal 4314, Lima 100
Teléfono: (511) 319-0000 Anexo: 2271 / 2542 **Telefax:** 482-4541
E-mail: duict@oficinas-upch.pe
Página Web: http://www.upch.edu.pe/vrinve/duict/

SECTOR SALUD

INSTITUTO NACIONAL DE SALUD

REPUBLICA DEL PERU

N°. 718-2011-DG-OGITT-OPE/INS

RESOLUCIÓN DIRECTORAL

Lima, 24 de Agosto del 2011

Visto, el expediente **N° 00010358-11**, presentado por **Asociación Civil Selva Amazónica**, mediante el cual solicita Autorización del protocolo de investigación, ensayo clínico titulado: **"MEJORAR EL CRECIMIENTO Y DESARROLLO EN LA INFANCIA TEMPRANA EN PAISES DE BAJOS RECURSOS A TRAVES DE UN PROGRAMA DE DESPARASITACION INCORPORADO EN LA ATENCION INTEGRADA DE SALUD INFANTIL"** según protocolo **11005**, patrocinado por **TRASHER RESEARCH FUND** y ejecutado por **ASOCIACION CIVIL SELVA AMAZONICA**;

CONSIDERANDO:

Que, el numeral XV del Titulo Preliminar de la Ley N° 26842 - Ley General de Salud, establece que el Estado promueve la investigación científica y tecnológica en el campo de la salud; asimismo, en su Artículo 28°, preceptúa que la investigación experimental con personas debe ceñirse a la legislación especial sobre la materia y a los postulados éticos contenidos en la Declaración de Helsinki y sucesivas declaraciones que actualicen los referidos postulados;

Que mediante el articulo primero del Decreto Supremo N° 034-2008-PCM se aprueba la calificación del Instituto Nacional de Salud como Organismo Público Ejecutor en concordancia con la Ley N° 29158; y de acuerdo al artículo 34° del Reglamento de la Ley del Ministerio de Salud, aprobado por Decreto supremo N° 013-2002-SA, tiene entre sus objetivos, la promoción, desarrollo y difusión de la investigación científica - tecnológica, como organismo de referencia nacional;

Que, el Reglamento de Organización y Funciones del Instituto Nacional de Salud, aprobado mediante Decreto Supremo N° 001-2003-SA, establece como objetivo del Instituto Nacional de Salud, entre otros, el desarrollo de investigaciones en salud para la prevención de riesgo, protección del daño, recuperación de la salud y rehabilitación de las capacidades de las personas;

Que, el Reglamento de Ensayos Clínicos, aprobado con D.S. N° 017-2006-SA y modificado por D.S. N° 006-2007-SA en sus artículos 4° y 5° establece que el Instituto Nacional de Salud es la autoridad encargada a nivel nacional, de velar por el cumplimiento del Reglamento y demás normas conexas que rigen la autorización y ejecución de los ensayos clínicos, a través de la Oficina General de Investigación y Transferencia Tecnológica;

Que, según documentos de visto con fecha 06 de abril del 2011, el ejecutor ASOCIACION CIVIL SELVA AMAZONICA S.A. solicitó Autorización para la realización del ensayo clínico;

Que habiéndose absuelto las observaciones emitidas según Carta s/n, recibida el 12 de julio del 2011, en respuesta a oficio N° 1043-2011-DG-OGITT-OPE/INS; y Carta N° 188-P-CD-2011-ACSA recibida

el 17 de agosto del 2011, en respuesta al oficio N° 1333-2011-DG-OGITT-OPE/INS de fecha 27 de julio del 2011;

De conformidad con el artículo 4° del Reglamento de Ensayos en el Perú, aprobado con Decreto Supremo 017-2006-SA y su modificatoria aprobada con Decreto Supremo 006-2007-SA;

SE RESUELVE:

Artículo 1° .- **AUTORIZAR** a **ASOCIACIÓN CIVIL SELVA AMAZÓNICA**, la realización del proyecto de investigación, ensayo clínico titulado **"MEJORAR EL CRECIMIENTO Y DESARROLLO EN LA INFANCIA TEMPRANA EN PAÍSES DE BAJOS RECURSOS A TRAVÉS DE UN PROGRAMA DE DESPARASITACIÓN INCORPORADO EN LA ATENCIÓN INTEGRADA DE SALUD INFANTIL"** según protocolo **11005**, patrocinado por **Thrasher Research Fund** y ejecutado por **ASOCIACIÓN CIVIL SELVA AMAZÓNICA**, la misma que tendrá vigencia de 12 meses a partir de la fecha.

Artículo 2°.- El ensayo clínico tendrá como centro de investigación a:

- Institución de Investigación: **ASOCIACION CIVIL SELVA AMAZONICA**
  Centro de Investigación: **RCI-129 ASOCIACION CIVIL SELVA AMAZONICA**
  Investigador Principal: **Dr. WILFREDO MARTIN CASAPIA MORALES**

Artículo 3°.- **ASOCIACIÓN CIVIL SELVA AMAZÓNICA**, queda en la obligación de:

- Presentar al Instituto Nacional de Salud informes de avance semestral durante la ejecución del Ensayo Clínico.
- Presentar al Instituto Nacional de Salud un informe al término del estudio y un informe final con los resultados y las conclusiones del ensayo clínico.
- Informar a la Oficina General de Investigación y Transferencia Tecnológica (OGITT) del Instituto Nacional de Salud todo Evento Adverso Serio.
- Informar y detallar en forma escrita los motivos de una suspensión y cancelación del ensayo clínico.
- Brindar las facilidades para la inspección de la ejecución del ensayo clínico por el personal de la Oficina General de Investigación y Transferencia Tecnológica (OGITT) del Instituto Nacional de Salud.

Artículo 4°.- Todos los materiales e insumos que se utilizarán para el desarrollo total del ensayo clínico no tienen un valor comercial, sino un valor nominal, y están contenidos en el anexo "Listado de Suministros a Utilizar en el Ensayo Clínico", que forma parte de la presente Resolución.

Artículo 5°.- Notificar la presente Resolución al recurrente.

Regístrese y comuníquese

[illegible] MÁXIMO MANUEL ESPINOZA SILVA
Director General
[illegible] de Investigación y Transferencia Tecnológica
Instituto Nacional de Salud

EC: 032-11
